# Identification of Potential Diagnoses Based on Immune Infiltration and Autophagy Characteristics in Major Depressive Disorder

**DOI:** 10.3389/fgene.2022.702366

**Published:** 2022-04-26

**Authors:** Ye Sun, Jinying Li, Lin Wang, Ting Cong, Xiuli Zhai, Liya Li, Haikuo Wu, Shouxin Li, Zhaoyang Xiao

**Affiliations:** ^1^ Department of Anesthesiology, The Second Affiliated Hospital of Dalian Medical University, Dalian, China; ^2^ Department of Anesthesiology, Inner Mongolia People’s Hospital, Hohhot, China

**Keywords:** MDD, GEO, immune infiltration, autophagy, depression subtypes

## Abstract

**Background:** Major depressive disorder (MDD) is a serious mental illness characterized by mood changes and high suicide rates. However, no studies are available to support a blood test method for MDD diagnosis. The objective of this research was to identify potential peripheral blood biomarkers for MDD and characterize the novel pathophysiology.

**Methods:** We accessed whole blood microarray sequencing data for MDD and control samples from public databases. Biological functions were analysed by GO and KEGG pathway enrichment analyses using the clusterprofile R package. Infiltrated immune cell (IIC) proportions were identified using the CIBERSORT algorithm. Clustering was performed using the ConsensusClusterPlus R package. Protein–protein interactions (PPI) were assessed by constructing a PPI network using STRING and visualized using Cytoscape software. Rats were exposed to chronic unpredictable mild stress (CUMS) for 6 weeks to induce stress behaviour. Stress behaviour was evaluated by open field experiments and forced swimming tests. Flow cytometry was used to analyse the proportion of CD8^+^ T cells. The expression of the corresponding key genes was detected by qRT–PCR.

**Results:** We divided MDD patients into CD8H and CD8L clusters. The functional enrichment of marker genes in the CD8H cluster indicated that autophagy-related terms and pathways were significantly enriched. Furthermore, we obtained 110 autophagy-related marker genes (ARMGs) in the CD8H cluster through intersection analysis. GO and KEGG analyses further showed that these ARMGs may regulate a variety of autophagy processes and be involved in the onset and advancement of MDD. Finally, 10 key ARMGs were identified through PPI analysis: RAB1A, GNAI3, VAMP7, RAB33B, MYC, LAMP2, RAB11A, HIF1A, KIF5B, and PTEN. In the CUMS model, flow cytometric analysis confirmed the above findings. qRT–PCR revealed significant decreases in the mRNA levels of Gnai3, Rab33b, Lamp2, and Kif5b in the CUMS groups.

**Conclusion:** In this study, MDD was divided into two subtypes. We combined immune infiltrating CD8^+^ T cells with autophagy-related genes and screened a total of 10 ARMG genes. In particular, RAB1A, GNAI3, RAB33B, LAMP2, and KIF5B were first reported in MDD. These genes may offer new hope for the clinical diagnosis of MDD.

## Introduction

Major depressive disorder (MDD) is the leading cause of the burden of mental health-related disease, affecting approximately 300 million people worldwide (Herrman et al., 2019). Currently, MDD is mainly diagnosed and treated on the basis of different symptoms and signs fitting to rigorous diagnostic categories, such as the Diagnostic and Statistical Manual of Mental Disorders, Fifth Edition (DSM-5) (Hasin et al., 2018). However, this diagnostic method has a certain degree of subjectivity, resulting in considerable limitations and errors in the diagnosis results. In the last several decades, some diagnostic biomarkers for MDD have been identified. However, none of these biomarkers achieve satisfactory specificity and sensitivity for clinical application (Kennis et al., 2020). One of the main causes of this dilemma is the lack of a thorough understanding of the pathophysiology and pathogenesis of depression, which complicates the diagnosis and treatment of depression.

Clinical and preclinical studies have suggested that the immune system is implicated in the pathophysiology of MDD (Dantzer et al., 2008; Wohleb et al., 2016). Changes in peripheral and central immune function are found in MDD patients (Medina-Rodriguez et al., 2018). The reduced circulating T cells and regulatory B cells indicate that the adaptive immune system malfunctions in MDD patients (Ahmetspahic et al., 2018). In addition, the ability of T cells in MDD patients to respond to stimuli is decreased, suggesting that T cells have an immunosuppressive phenotype in MDD (Herbert and Cohen, 1993; Zorrilla et al., 2001; Irwin and Miller, 2007). The above results have motivated a search for biomarkers of immune status that can be used to stratify MDD cases. Blood is an easily obtainable tissue used to analyse immune biomarkers, while collecting brain tissue is inconvenient. Among the blood biomarkers associated with MDD immune pathogenesis, the central nervous system (CNS) immune status has been identified to be related to or result from peripheral immune status. Furthermore, gene transcription detected within human blood samples has been increasingly suggested to be related to the transcript levels detected in numerous additional body systems, such as the CNS (Wittenberg et al., 2020). Therefore, the development of immune biomarkers and accompanying diagnoses could be used to guide new therapeutic methods.

Previous studies have demonstrated that autophagy is associated with the immune response, cytokine production and secretion, and inflammasome activation in many physiological and pathological processes (Ali et al., 2020; Lee et al., 2020). Autophagy is a self-degradation process that maintains cellular homeostasis by autophagosome formation (Onorati et al., 2018). Widely used antidepressants such as amitriptyline and fluoxetine activate autophagy in hippocampal neurons (Gulbins et al., 2018). Autophagy marker levels are increased after treating cells with antidepressants (Gassen et al., 2015). Although the function of autophagy in MDD has been reported, the intersection between immune infiltration and autophagy affecting the MDD process is unknown.

In this study, we studied immune status biomarkers that could be used for MDD stratification. Briefly, we identified IIC-based MDD subtypes by CIBERSORT and consensus clustering using the GEO database. Marker genes of the subtypes were also obtained and functionally enriched to reveal potential functions. In summary, this study identified new subtypes of depression that exceed the current diagnostic limits, which may help identify individuals who are most likely to benefit from targeted therapies.

## Methods

### Data Collection

Two gene expression profiles of whole-blood samples from MDD patients and healthy donors were obtained from the Gene Expression Omnibus (GEO) database. The GSE98793 dataset included 128 MDD patients and 64 healthy controls. Twenty-one MDD samples, eight bipolar disorder samples (BDI), and 24 healthy donors were included in the GSE39653 dataset. Eight bipolar disorder samples were excluded from this study. The GSE98793 dataset was used as a training set, and the GSE39653 dataset was used as a testing set. In addition, a total of 222 autophagy-related genes (ARGs) were obtained from the Human Autophagy Database (HADb).

### Elimination of the Batch Effect

Batch effects are subgroups of measurements with different qualitative behaviours under different conditions independent of the biological or scientific variables under study (Leek et al., 2010). In this study, the GSE98793 dataset used as the training set was composed of two batches of sequencing data. Therefore, we performed a batch effect reduction exercise on the two batches of sequencing data in this dataset using the removeBatchEffect function of the limma package in R. Principal component analysis (PCA) was used to demonstrate the distribution of samples before and after the removal of the batch effect (Supplementary Figure S1). Moreover, to maximally increase the reliability of the validation results in the GSE39653 dataset, we narrowed the batch effect between the GSE39653 dataset and the GSE98793 dataset using the same method (Supplementary Figure S2).

### Assessment of Immune Infiltration

CIBERSORT (an analytical tool from the Alizadeh Lab and Newman Lab to impute gene expression profiles and provide an estimation of the abundances of member cell types in a mixed cell population, using gene expression data) precisely quantified the different types of immune cells in each sample (Newman et al., 2015). In the current study, LM22 (the original CIBERSORT gene signature file) was utilized to measure the fractions of immune cells from MDD and healthy samples. Samples with a *p*-value less than 0.05 suggested that the fractions inferred by the algorithm are accurate (Ali et al., 2016) and could be used for further analysis. In the present study, all samples from the GSE98793 (128 MDD and 64 healthy samples) and GSE39653 (21 MDD samples and 24 healthy samples) datasets were satisfied with the CIBERSORT-*P* < 0.05 criterion. The sum of the immune cell type fractions assessed for each sample was 1, which could be regarded as cell fractions compared between immune cell types and datasets. Based on the IIC abundance profiles in the GSE98793 and GSE39653 datasets, a Wilcoxon rank-sum test was performed to analyse the comparison of IIC proportions between the healthy and MDD groups. Next, the correlation between IICs was analysed by Pearson correlation analysis. In addition, immune cytolytic activity was another criterion used to measure immune infiltration following the geometric mean of granzyme A and perforin 1 (Connor et al., 2017).

### Unsupervised Consensus Clustering

Based on the fraction profile of IICs obtained by CIBERSORT, unsupervised consensus clustering was performed using the R package ConsensusClusterPlus (Wilkerson and Hayes, 2010) on MDD samples in the training set (*n* = 128) and validation set (*n* = 21) to identify MDD immune subtypes. Notably, 128 MDD samples in the training set were from the GSE98793 dataset; 21 MDD samples in the validation set were taken from the GSE39653 dataset. All MDD samples passed the CIBERSORT-P < 0.05 test. The K-means algorithm was used for 1,000 resampling iterations to ensure the stability of the consensus clustering. The optimal cluster number was determined using the consensus matrix (CM) and CDF curves of the consensus score (Zhou et al., 2020).

### Identification of Marker Genes in Different Clusters

The Limma R package (version 3.42.2) based on t-test statistical significance was applied to identify the marker genes, which were defined as significant by an adjusted *p*-value less than 0.05 (Tusher et al., 2001). The gene expression level acted as the primary variable for comparison between three clusters in the GSE98793 dataset. Here, marker genes of subgroups refer to genes that were overexpressed or repressed in the target subgroup compared to other subgroups, For example, marker genes for the T cell CD8H subpopulation were defined as genes that were significantly up- and down-regulated in the T cell CD8H subpopulation compared to the T cell CD8L + control group (adj. *p* < 0.05).

### Enrichment Analysis

Annotations and classifications of genes were carried out by GO (Zhang et al., 2017) according to biological process (BP), molecular function (MF), and cellular component (CC) (Ashburner et al., 2000; Consortium, 2006). KEGG interpreted molecular interactions, reactions and relationship networks (Kanehisa and Goto, 2000). Herein, the clusterPrifiler R package was applied for GO enrichment and KEGG pathway analyses of the identified marker genes and ARMGs with a threshold *p*-value < 0.05.

### Formation of Protein–Protein Interaction Network

STRING (http://string-db.org/) (von Mering et al., 2003), a tool for interacting gene retrieval, is a biological database for predicting PPI information. The marker genes were mapped to STRING for interaction evaluation, and a confidence level >0.9 was considered significant. Subsequently, we selected nodes with a degree ≥10 to construct a subnetwork and identified marker genes belonging to ARGs. Then, Cytoscape (Shannon et al., 2003) was utilized to construct PPI networks.

### Identification of Key Autophagy-Related Marker Genes

Overlap analysis was performed to identify common elements between marker genes of T cell CD8H and 222 ARGs, which were defined as ARMGs. the overlap analysis was performed by the Jvenn online analysis tool (http://jvenn.toulouse.inra.fr/app/example.html). Briefly, the list of T cell CD8H-marker genes and 222 ARGs was uploaded to the Jvenn tool to identify the overlapping elements in the two gene lists. Subsequently, the same method was used to identify ARMGs in the list of T cell CD8H-marker genes with degree ≥10, which were considered as key ARMGs.

### Animals

The Animal Care and Use Committee of Dalian Medical University (Dalian, China) authorized the animal experiments in this study, which were performed in accordance with the National Institute of Health Guide. The Experimental Animal Centre of Dalian Medical University provided Sprague–Dawley rats (200–250 g), which were housed in a specialized pathogen-free facility. Rats were maintained under a stringent 12-h light and 12-h dark cycle and had free access to water and food. All experimental protocols were approved by Animal Experiment Ethics Committee of Dalian Medical University (Reference number: AEE19087).

### Chronic Unpredictable Mild Stress Procedure

Chronic unpredictable stress stimulation was used to establish the chronic stress model. According to our previous research, we adopted an improved stress stimulation method (Zhang et al., 2021). All animals were allowed to adapt to the living environment for 1 week. During this experiment, the rats were placed in a cage in a separate room without interference. Animals received one of the following stressors once a day for 6 weeks: restraint for 1 h, cold water swimming (4°C) for 5 min, warm water swimming (45°C) for 5 min, food or water deprivation for 24 h, tail clamp for 1 min, overnight illumination for 12 h, damp bedding (200 ml water in 100 g sawdust bedding) for 24 h, and cage tilting (45°C) for 24 h. To prevent habituation and provide an unpredictable characteristic for stressors, all the stressors were randomly applied within 1 week and repeated throughout the 6-week experiment. At the same time, animals in the control group maintained a normal feeding schedule.

### Open Field Test

The rats’ behaviour in the open field was examined using the Super Maze V2.0 Animal Behaviour Video Analysis System (Shanghai Xinruan Technology Co., Ltd., China). Rats were recorded for 5 min. The box (50 cm × 50 cm × 40 cm) included black inner walls with a bottom containing a 25-square lattice for analysis. The light was set at approximately 40 LX, while the laboratory noise in the background was set <65 dB. The total distance travelled and the number of crossing in central area of the open field were analysed. The total motor distance reflected the general locomotor activity of rats. Exploratory behavior and anxiety were measured by the number of crossing in central area. The cages were cleaned with 75% alcohol after the test to avoid odour affecting tests with subsequent animals.

### Forced Swimming Test

The FST was used to assess motivated behavior. The FST was performed according to previously reported methods (Zhang et al., 2021). Briefly, a rat was placed into a transparent cylinder filled with water (25°C). The rats were considered immobile as long as they remained passively floating in the water with their noses out of the water. All rats were forced to swim for 6 min: the first 2 min were the adaptation stage, and the duration of immobility was recorded during the last 4 min.

### Flow Cytometric Analysis

The peripheral blood samples from all the groups at 6 weeks were collected in EDTA tubes (BD Vacutainer, United States). The samples were blocked with fuorescence conjugated antibodies [anti-mouse CD3 antibody and anti-mouse CD8 antibody (BD Biosciences, United States)] for 20 min in the dark, at room temperature. After washing, the samples were lysed using the lysing solution for 10 min and washed and suspended in PBS. A minimum of 10,000 stained nucleated cells per tube were acquired using a flow cytometer (BD Biosciences, United States) and analyzed using FlowJo 7.6 software (TreeStar, Inc.).

### Quantitative Real-Time Polymerase Chain Reaction

All whole-blood samples lysed with TRIzol LS reagent (Invitrogen, United States), and total RNA was isolated following the manufacturer’s instructions. Then the concentration and purity of the RNA solution were quantified using a NanoDrop 2000FC-3100 nucleic acid protein quantifier (Thermo Fisher Scientific, United States). The extracted RNA was reverse-transcribed to cDNA using the TranScript Top Green qPCR kit (TransGen Biotech, China) prior to qRT-PCR. The qRT-PCR reaction consisted of 2 µl of reverse transcription product, 10 µl of 2 × SYBR Qpcr Master Mix (Bori, China), and 0.4 µl each of forward and reverse primer. PCR was performed in a BIO-RAD CFX96 Touch TM PCR detection system (Bio-Rad Laboratories, Inc., United States) under the following conditions: initial denaturation at 95°C for 30 s, followed by 40 cycles that each involved incubation at 95°C for 5 s, 60°C for 30 s, followed by 65°C for 5 s, and 95°C for 5 s. The forward primer of *Rab1a* was “TCC​TCC​CCT​TCC​TTT​ACC​CG”. The reverse primer of *Rab1a* was “CCG​TAT​ACG​TGT​CAT​CCG​CAA”. The forward primer of *Gnai3* was “TGA​GGA​CGA​GGA​AAT​GAA​CCG​A”. The reverse primer of *Gnai3* was “TGC​ACG​TTT​TTG​GTG​TCA​GTG”. The forward primer of *Rab33b* was “ATG​GAG​AAC​GCA​TTA​AGA​TCC​AGT”. The reverse primer of *Rab33b* was “AAG​CTG​GCC​ATG​TTG​GTC​AT”. The forward primer of *Lamp2* was “GGC​TAA​TGG​CTC​AGC​TTT​CCA”. The reverse primer of *Lamp2* was “TGA​TGG​CGC​TTG​AGA​CCA​AT”. The forward primer of *Kif5b* was “ACG​AGT​CTG​AAG​TGA​ACC​GC”. The reverse primer of *Kif5b* was “ATG​CAT​AAG​GCT​TGG​ACG​CA”. The forward primer of *Gapdh* was “ATG​CCG​CCT​GGA​GAA​ACC”. The reverse primer of *Gapdh* was “GCA​TCA​AAG​GTG​GAA​GAA​TGG”. All primers were synthesized by Sangon biotech (Sangon biotech, China). The GAPDH gene served as an internal control, and the relative expression of five genes was determined using the 2^−Δ^ (ΔCT) method. Statistical differences of *Rab1a, Gnai3, Rab33b, Lamp2, Kif5b* genes between control and CUMS samples were detected by unpaired t-tests, using GraphPad Prism 7 (GraphPad Software, United States), and the level of statistical significance was tested and represented as * for *p* < 0.05.

### Statistical Analysis

R software (version 3.6.0) was used for statistical analyses. The Wilcoxon rank-sum test assessed the different ARMGs in the CD8^+^ T cell cluster and other clusters. SPSS 20.0 (IBM, NY) and GraphPad Prism 7 (GraphPad, CA) software were used for statistical analysis. The data are presented as the mean ± SEM. One-way ANOVA and Tukey’s multiple comparison post hoc tests were conducted to determine differences between individual groups. Student’s t-test was employed to identify differences between two groups. A value of *p* < 0.05 was considered statistically significant.

## Results

### Analysis of Immune Landscape in the GSE98793 Dataset

**FIGURE 1:**
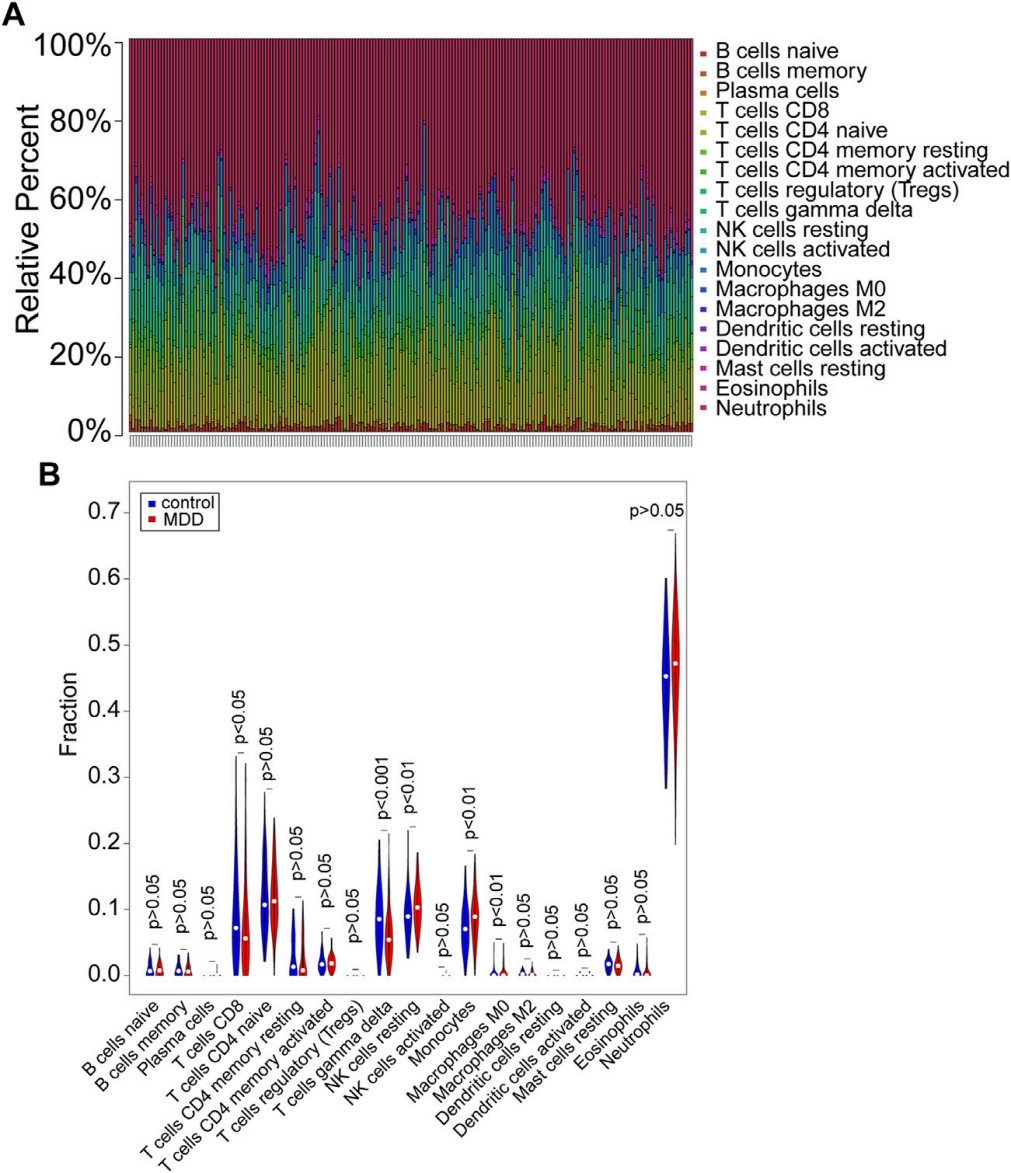
Evaluation and visualization of immune cell infiltration in the GSE98793 dataset. **(A)** The proportion of immune cells in the MDD and control groups. Immune cells were represented by different colors. The vertical bars represented different samples. **(B)** The violin plot exhibited the differences in CIBERSOFT immune cell fractions between MDD and controls. MDD (*n* = 128): major depressive disorder. Control (*n* = 64): healthy donors. CIBERSOFT: an analytical tool from the Alizadeh Lab and Newman Lab to impute gene expression profiles and provide an estimation of the abundances of member cell types in a mixed cell population, using gene expression data. *p* < 0.05 was considered statistically significant.

CIBERSORT (*p* < 0.05) was used to calculate the differential IICs between 128 MDD samples and 64 controls following the Wilcoxon rank-sum test from GSE98793 (Figures 1A,B, Supplementary Table S1). The number of resting NK cells (*p* < 0.01), monocytes (*p* < 0.01) and M0 macrophages (*p* < 0.01) was increased in MDD patients, while the other immune cell types, including CD8^+^ T cells (*p* < 0.05) and gamma delta T cells (*p* < 0.001), were decreased (Figures 1A,B, Supplementary Table S1). These results indicated different IIC proportions in MDD samples and controls from the GSE98793 dataset.

We further explored the relationship of IICs by correlation analysis. The results showed a negative relationship between T cells CD8 and T cells CD4 memory resting (cor = −0.48, *p* < 0.001) and Neutrophils (cor = −0.42, *p* < 0.001); B cells naïve had a negative correlation with B cells memory (cor = −0.46, *p* < 0.001) and positively correlated with T cells CD4 naïve (cor = 0.31, *p* < 0.001); Monocytes negatively correlated with Macrophages M2 (cor = −0.43, *p* < 0.001) (Supplementary Figure S3A). Furthermore, Supplementary Figure S3B demonstrated that cytolytic activity was positively correlated with T cell gamma delta (cor = 0.51, *p* < 0.001), T cell CD8 (cor = 0.37, *p* < 0.001), and NK cell resting (cor = 0.33, *p* < 0.001), whereas it was negatively correlated with Neutrophils (cor = −0.51, *p* < 0.001).

### Identification and Validation of Major Depressive Disorder Subtypes Based on Infiltrated Immune Cell

**FIGURE 2:**
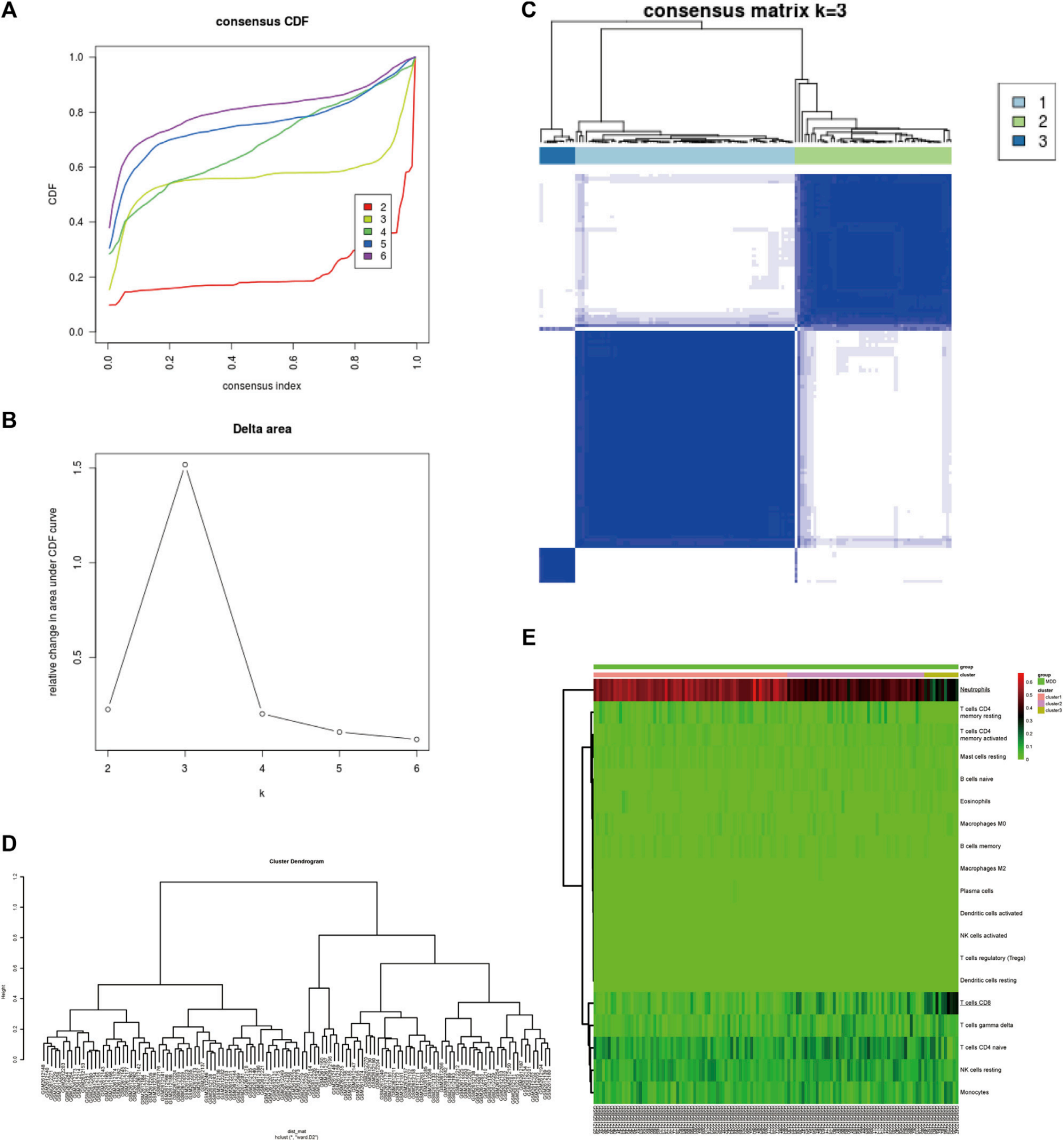
Identification of MDD subtypes based on IICs in the GSE98793 dataset. **(A)** The cumulative distribution function (CDF) curves in consensus cluster analysis. Consensus scores for different subtype numbers (k = 2–6) are presented. **(B)** The relative change in area under the CDF curve for k = 2–6. **(C)** The consensus matrix of all samples was distributed into three clusters at k = 3. **(D)** The cluster dendrogram among all samples. **(E)** Heatmaps of IICs proportions of each cluster. The higher and lower expressed genes were shown in red and green, respectively, and genes with the same expression level in black. IICs, infiltrated immune cells; MDD (*n* = 128), major depressive disorder.

To identify subgroups of samples, immune infiltration MDD samples were selected based on a consensus clustering analysis using the Consensus Cluster Plus package. Based on the CIBERSORT algorithm, 128 MDD samples with *p* < 0.05 were mined from the GSE98793 cohort and analysed by consensus clustering using the R package Consensus Cluster Plus. The results revealed that the relative change in the area under the cumulative distribution function (CDF) curve was significantly reduced when k = 3, which implied that the sample clusters were stable and robust (Figures 2A–C). Among them, Cluster one contained 68 MDD samples, Cluster two included 48 MDD samples, and Cluster three comprised 12 MDD samples. Similarly, the cluster dendrogram also exhibited three individualized clusters among all MDD samples (Figure 2D). The heatmap showed that CD8^+^ T cells and neutrophils exhibited significantly different proportions among the three clusters, including a high proportion of CD8 or neutrophils, an intermediate proportion of CD8 or neutrophils, and a low proportion of CD8 or neutrophil clusters (Figure 2E), indicating differentially expressed IICs between MDD samples, especially CD8^+^ T cells and neutrophils.

**FIGURE 3:**
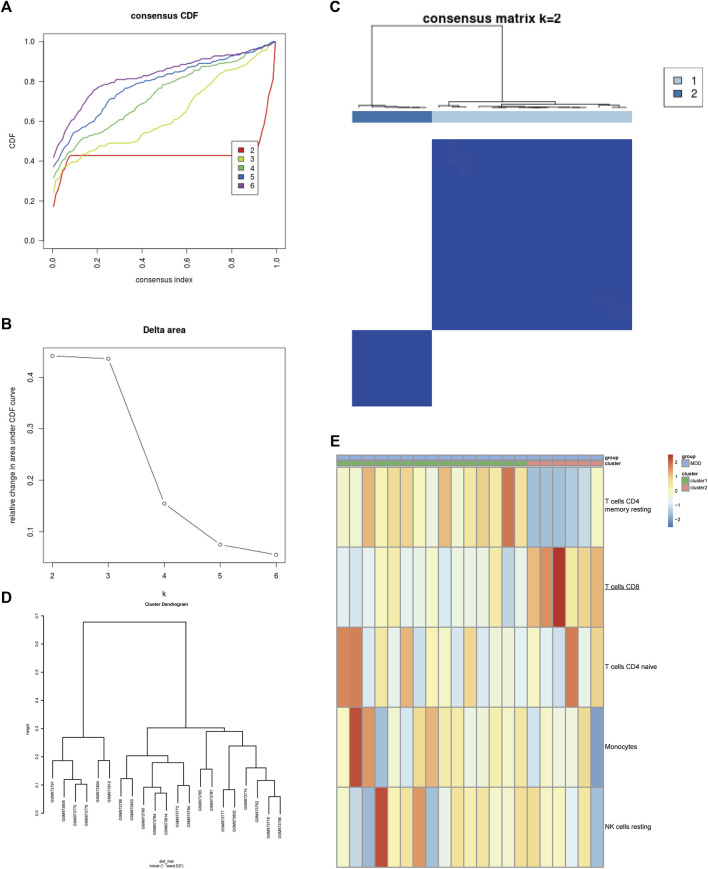
Validation of the clustering in the GSE39653 dataset. **(A)** Unsupervised clustering with the k-means algorithm of entire samples. **(B)** Relative change in area under CDF curve for k = 2–6. **(C)** The optimal number of consensus clustering. **(D)** The cluster dendrogram among all samples. **(E)** Comparison of IIC proportions of the top five MAD (median absolute deviation) values among MDD clusters. Red indicates relative upregulation of gene expression; blue indicates the relative downregulation of gene expression; light yellow indicates no significant change in gene expression. MDD (*n* = 21), major depressive disorder; IICs, infiltrated immune cells.

Next, the GSE39653 dataset was used to validate the clustering in Figure 2. The fractions of IICs among 45 samples (24 controls and 21 MDD samples) from the GSE39653 dataset were evaluated, and the results showed that B cell memory (*p* < 0.01) and CD8^+^ T cells (*p* < 0.05) were significantly lower in MDD samples than in the control group; naive B cells (*p* < 0.05) were significantly overexpressed in MDD samples (Supplementary Figure S4, Supplementary Table S2). The consensus clustering results revealed that MDD samples were divided into two clusters (Figures 3A–D). Among them, Cluster one contained 15 MDD samples and Cluster two included six MDD samples. All samples were divided into high and low proportions of CD8^+^ T cells based on IIC fractions (Figure 3E).

Based on a previous study (Figure 2), MDD was finally divided into two subtypes—a high proportion of CD8^+^ T cells (T cells CD8H; *n* = 12) and a low proportion of CD8^+^ T cells (T cells CD8L; *n* = 116)—for further research.

### Identification of Marker Genes in the T Cell CD8H Cluster and T Cell CD8L Cluster

**FIGURE 4:**
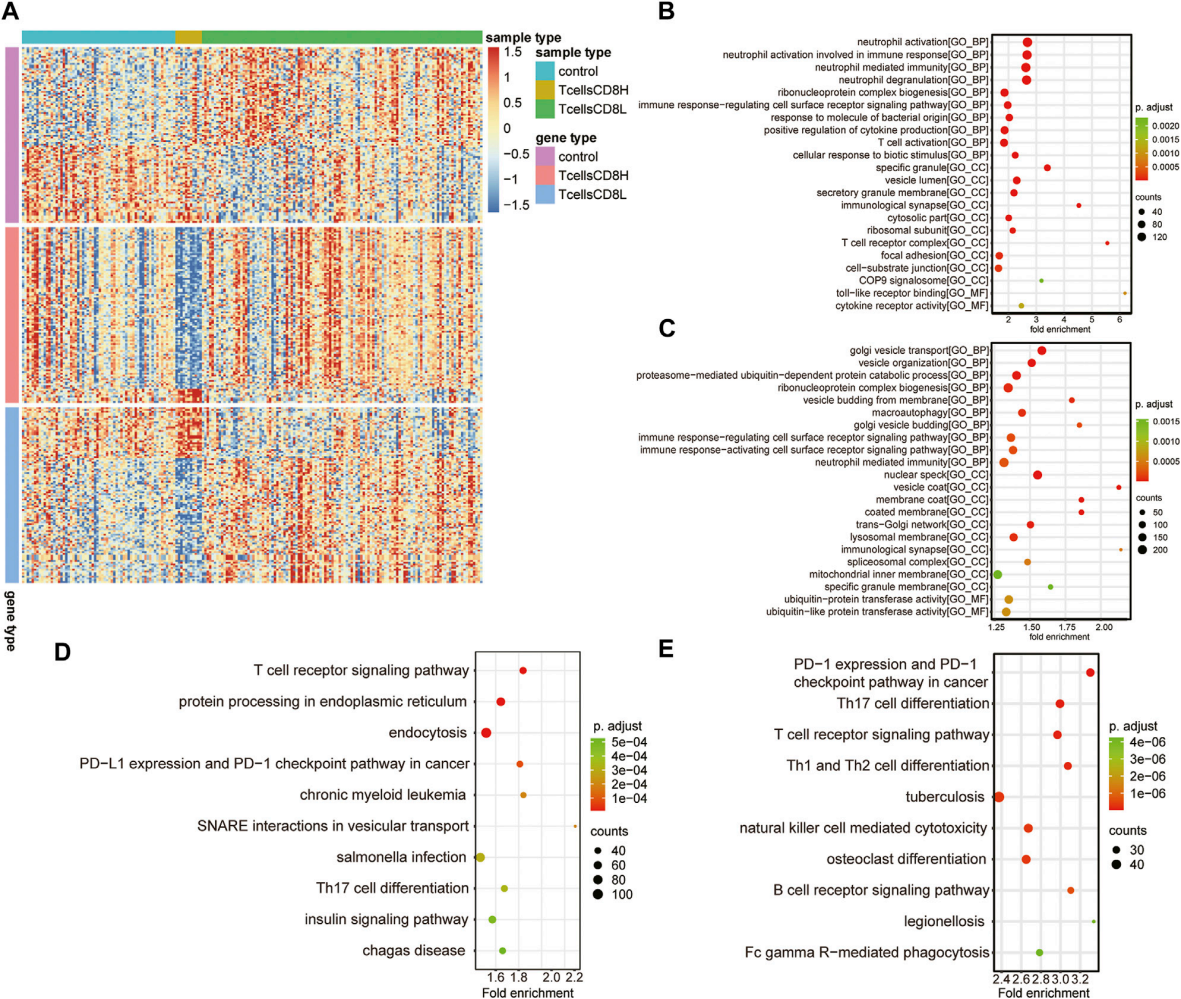
Potential functions of marker genes in the T cell CD8H cluster and T cell CD8L cluster. **(A)** The expression heatmap of the top 100 marker genes (sorted according to |log_2_ Fold change|) for the normal group, T cells CD8H cluster, and T cells CD8L cluster. Red indicates relative upregulation of gene expression; blue indicates the relative downregulation of gene expression; light yellow indicates no significant change in gene expression. **(B,C)** The dotplot of the GO enriched BP, MF, and CC terms in marker genes of the T cell CD8L **(B)** and T cell CD8H **(C)** clusters. **(D,E)** Significant KEGG pathways of marker genes in the T cell CD8H **(D)** and T cell CD8L **(E)** clusters. The larger the circle, the more genes it contained; conversely, the smaller the circle, the fewer genes it contained. The color of the circle is correlated with the *p* Value. The lower the *p* Value is, the closer it is to the red value. The higher the *p* Value is, the closer it is to the green value. GO, Gene Ontology; BP, biological process; CC, cellular component; MF, molecular function; KEGG, Kyoto Encyclopedia of Genes and Genomes. Control: healthy donors (*n* = 64). T cell CD8H: a high proportion of CD8^+^ T cells (*n* = 12). T cell CD8L: a low proportion of CD8^+^ T cells (*n* = 116).

To explore the differences between subclusters and identify the biomarkers of each cluster, the marker genes were determined using the Limma R package. A total of 6,159 marker genes, including 3,728 upregulated and 2,431 downregulated genes, were screened in the T cell CD8H cluster (compared to the T cell CD8L + control group). The top five genes with the largest fold change were CTB-167B5.2, TMEM55A, RP11–488L18.10, OLFM4, and RAB33B (Figure 4A; Table 1). Moreover, 2,423 marker genes, including 1,059 upregulated and 1,346 downregulated marker genes, were identified in the T cell CD8L cluster (compared to the T cell CD8H + control group), including LCN2, CNTNAP3B, DEFA4, CEACAM8, and OLFM4, which were the five genes with the largest fold change (Figure 4A; Table 1). One hundred thirty-six marker genes (56 upregulated genes and 80 downregulated genes) were identified in the normal group (compared to the T cell CD8H + T cell CD8L group, Figure 4A), but this study did not focus on these genes.

**TABLE 1:** Top 10 marker genes in two clusters.

Gene symbol	Cluster	Log FC	AveExpr	t	*p*-value	Adj. *p*-value	B
CTB-167B5.2	TcellsCD8H	−1.6757872	8.422562	−7.739781	*p* < 0.001	*p* < 0.001	20.5402649
TMEM55A	TcellsCD8H	−1.6712836	6.246543	−6.538500	*p* < 0.001	*p* < 0.001	14.0320453
RP11-488L18.10	TcellsCD8H	−1.6068182	6.966996	−5.322798	*p* < 0.001	*p* < 0.001	8.1650660
OLFM41	TcellsCD8H	−1.4794778	6.931726	−2.760569	*p* < 0.01	*p* < 0.05	−0.9998805
RAB33B	TcellsCD8H	−1.4475769	6.942980	−5.648441	*p* < 0.001	*p* < 0.001	9.6551374
LCN2	TcellsCD8L	0.7106662	8.076157	3.905290	*p* < 0.001	*p* < 0.01	2.5653464
CNTNAP3B	TcellsCD8L	0.7758796	6.827838	5.073552	*p* < 0.001	*p* < 0.001	7.0221153
DEFA4	TcellsCD8L	0.8900738	7.326248	4.246896	*p* < 0.001	*p* < 0.01	3.7715552
CEACAM8	TcellsCD8L	0.9371861	8.643687	4.441836	*p* < 0.001	*p* < 0.01	4.4969027
OLFM4	TcellsCD8L	1.0600829	6.931726	3.844797	*p* < 0.001	*p* < 0.01	2.3605874

### Functional Enrichment Analysis of Marker Genes in the T Cell CD8H Cluster and T Cell CD8L Cluster

All of the marker genes, as previously described, were analysed for their potential function in the Gene Ontology resource (GO; http://geneontology.org) and Kyoto Encyclopedia of Genes and Genomes (KEGG) pathways. GO terms in different GO categories (BP, MF, and CC) showed that T cell CD8L marker genes were mainly related to immune cell activation (neutrophil and T cell activation) (Figure 4B). Meanwhile, the marker gene CD8H of T cells was significantly associated with immune (immune response-regulating cell surface receptor signalling pathway), macroautophagy, and posttranscriptional modification processes (ribonucleoprotein complex biogenesis) (Figure 4C).

Furthermore, KEGG pathway analysis found that T cell CD8H marker genes were mainly involved in the T cell receptor signalling pathway, endoplasmic reticulum protein processing, and endocytosis (Figure 4D), while T cell CD8L marker genes were enriched in Th17 cell differentiation and the T cell receptor signalling pathway (Figure 4E). Notably, we found that the marker genes of the T cell CD8H cluster were enriched in terms related to autophagy, such as “macroautophagy”. Therefore, we focused on the T cell CD8H cluster, which was enriched in many autophagy-related terms.

### Analysis of Autophagy-Related Marker Genes in the T Cells CD8H Cluster

**FIGURE 5:**
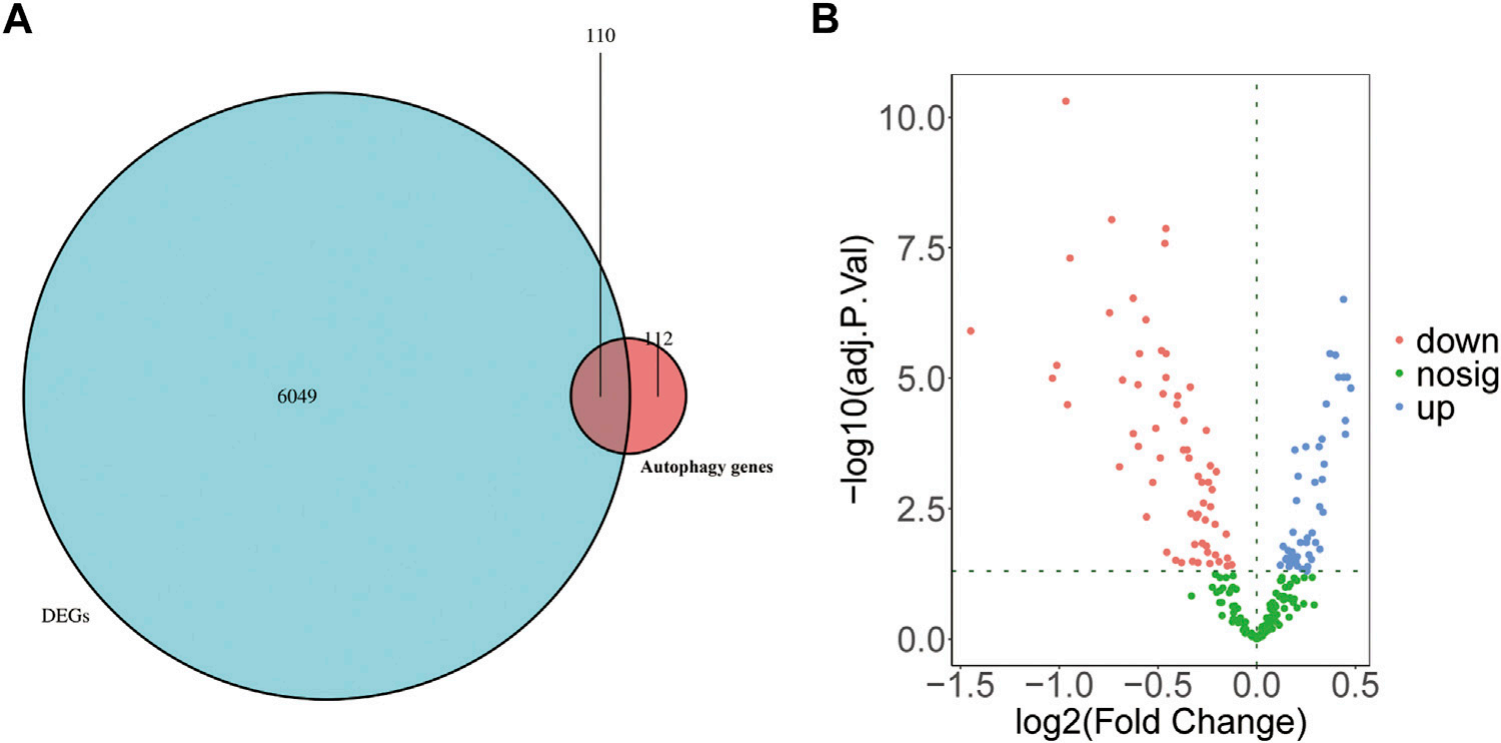
Identification of ARMGs in the T cell CD8H cluster. **(A)** Venn diagram showed the intersecting genes (denoted as ARMGs) from marker genes of T cell CD8H cluster (denoted as DEGs) and ARGs of HADb. Blue-Green area: marker genes of T cell CD8H cluster; red area: ARGs of HADb; cross area: genes expressed in both gene sets. **(B)** Volcano plot of the 110 ARMGs in the T cell CD8H cluster. Blue: upregulation with adj. *p* < 0.05; red: downregulation with adj. *p* < 0.05; green: unchanged genes. ARMGs, autophagy-related marker genes; DEG, differentially expressed gene; ARGs, autophagy-related genes; HADb, Human Autophagy Database.

To understand the possible role of ARGs in the T cell CD8H clusters in MDD patients, we performed the following analyses. First, 222 ARGs integrated from the HADb database intersected with 6,159 marker genes identified in the T cell CD8H cluster (Figure 5A). The Venn diagrams and volcano plots in Figures 5A,B show 47 upregulated and 63 downregulated ARMGs (*p* < 0.05). Then, the heatmap displayed the expression pattern of the ARMGs between T cells CD8H and other clusters (Supplementary Figure S5A). The PCA based on the different clusters revealed a distinguishing outcome, which is shown in Supplementary Figure S5B. These findings showed that 110 of 222 ARGMs in the HADb database were differentially expressed in T cell CD8H subtypes, suggesting that autophagy-related genes might play a role in T cell CD8H subtypes.

### Functional Enrichment Analysis of Autophagy-Related Marker Genes in the T Cell CD8H Cluster

**FIGURE 6:**
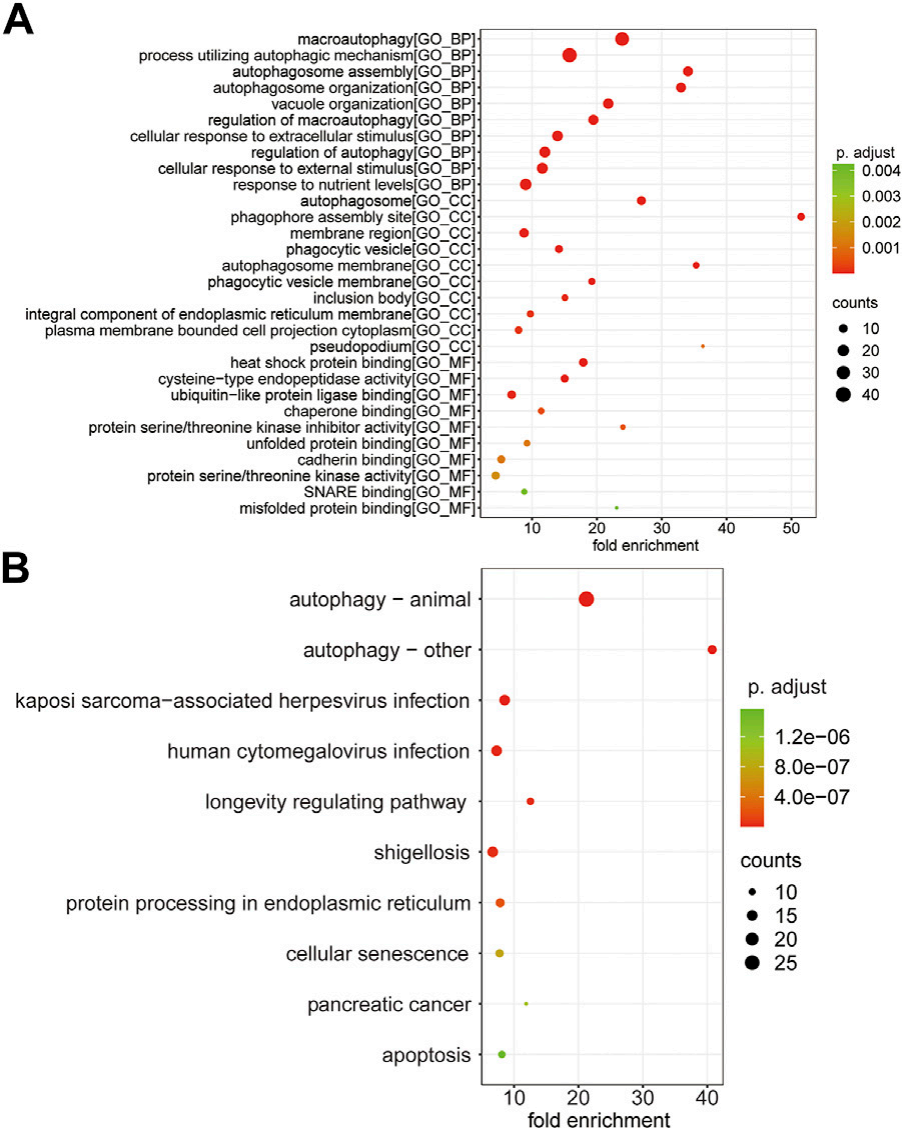
Functional analysis of 110 ARMGs in the T cell CD8H cluster. **(A)** GO enrichment result of ARMGs. The x-axis label represents fold enrichment and y-axis label represents GO terms. **(B)** Top 10 significantly enriched KEGG pathway in ARMGs. Y-axis label represents pathway, and X-axis label represents fold enrichment. Size and color of the bubble represent amount of ARMGs enriched in term and enrichment significance, respectively. The larger the circle, the more genes it contained; conversely, the smaller the circle, the fewer genes it contained. The color of the circle is correlated with the *p* Value. The lower the *p* Value is, the closer it is to the red value. The higher the *p* Value is, the closer it is to the green value. GO, Gene Ontology; KEGG, Kyoto Encyclopedia of Genes and Genomes; ARMGs, autophagy-related marker genes. “BP” stands for “biological process”, “CC” stands for “cellular component” and “MF” stands for “molecular function”.

GO enrichment analysis was performed following the 110 ARMGs, including the BP, CC, and MF categories. The results indicated that macroautophagy, processes utilizing autophagic mechanisms, and autophagosome assembly were enriched terms in the BP category. In the CC category, 110 ARMGs were considerably enriched in terms of the autophagosome, phagophore assembly site, and membrane region. In the MF category, those ARMGs had obvious enrichment in terms of heat shock protein binding, cysteine-type endopeptidase activity, and ubiquitin-like protein ligase binding (Figure 6A, Supplementary Table S3).

KEGG pathway analysis suggested that ARMGs had visible enrichment in “Autophagy–animal” and “Autophagy–other”. In addition, the “Apoptosis” pathway was enriched in ARMGs, which was potentially implicated in the development and progression of MDD (Figure 6B, Supplementary Table S4).

### Screening of the Key Autophagy-Related Marker Genes

To investigate the interactions between 6,159 marker genes of the T cell CD8H cluster, we obtained a PPI network containing 566 nodes and 2,299 edges after presenting discrete individual proteins in STRING with an interaction score >0.9 (Supplementary Figure S6A). Among them, UBE2D1 (degree = 52), PIK3CA (degree = 41), and EP300 (degree = 40) were the top three proteins in terms of degree value, indicating that these proteins had tight interactions with numerous other marker gene proteins. We screened 170 marker genes with a degree ≥10 to further identify important ARGs and visualized a PPI subnetwork containing 1,363 interactions by Cytoscape (Supplementary Figure S6B). We found that RAB1A (degree = 30), RAB11A (degree = 26), VAMP7 (degree = 17), GNAI3 (degree = 16), PTEN (degree = 14), MYC (degree = 13), HIF1A (degree = 12), RAB33B (degree = 11), KIF5B (degree = 11), and LAMP2 (degree = 10) were ARGs, which were considered key ARMGs (Table 2).

**TABLE 2:** Implications of the 10 key ARMGs.

Gene symbol	Full name	Implications
RAB1A	Ras-Related Protein Rab-1A	Rab1a is the small G protein that regulates vesicle transport from endoplasmic reticulum to and through Golgi. Rab1a overexpression can increase the expression level of TLR4 Song et al. (2020). The association between RAB1A and MDD has not been reported
GNAI3	G Protein Subunit Alpha I3	Signaling is mediated via effector proteins, such as adenylate cyclase. Inhibits adenylate cyclase activity, leading to decreased intracellular cAMP levels Kimple et al. (2009). The association between GNAI3 and MDD has not been reported
VAMP7	Vesicle Associated Membrane Protein 7	Involved in the targeting and/or fusion of transport vesicles to their target membrane during transport of proteins from the early endosome to the lysosome. Vamp7 might provide insight into treatment of MDD Li et al. (2019)
RAB33B	Ras-Related Protein Rab-33B	Protein transport. Acts, in coordination with RAB6A, to regulate intra-Golgi retrograde trafficking. It is involved in autophagy, acting as a modulator of autophagosome formation. The association between RAB33B and MDD has not been reported
MYC	MYC Proto-Oncogene	MYC is somehow involved in depressed suicide Zeng et al. (2020)
LAMP2	Lysosomal Associated Membrane Protein 2	Plays an important role in chaperone-mediated autophagy, a process that mediates lysosomal degradation of proteins in response to various stresses and as part of the normal turnover of proteins with a long biological half-live. The association between LAMP2 and MDD has not been reported
RAB11A	Ras-Related Protein Rab-11A	Antidepressants AMPH leads to an increase of NET (The norepinephrine transporter is a major target for medications used for the treatment of depression) in a Rab11-dependent manner Matthies et al. (2010)
HIF1A	Hypoxia Inducible Factor 1 Subunit Alpha	altered expression of HIF-1 and its target genes mRNA in peripheral blood cells are associated-mainly in a state-dependent manner-with mood disorders (especially with MDD). In addition, altered expression of HIF-1 and its target genes may be associated with the pathophysiology of depression Shibata et al. (2013)
KIF5B	Kinesin Family Member 5B	Syntabulin acts as a KIF5B motor adaptor and mediates anterograde transport of presynaptic cargoes and mitochondria, presynaptic assembly, and activity-induced plasticity Ma et al. (2009). The association between KIF5B and MDD has not been reported
PTEN	Phosphatase And Tensin Homolog	PTEN serves as a key mediator in chronic stress-induced neuron atrophy as well as depression-like behaviors, providing molecular evidence supporting the synaptic plasticity theory of depression Chen et al. (2021), Wang et al. (2021)

### The Proportion of CD8^+^ T Cells and the Level of Corresponding Key Genes in the Peripheral Blood of Rats in the Chronic Unpredictable Mild Stress Model

**FIGURE 7:**
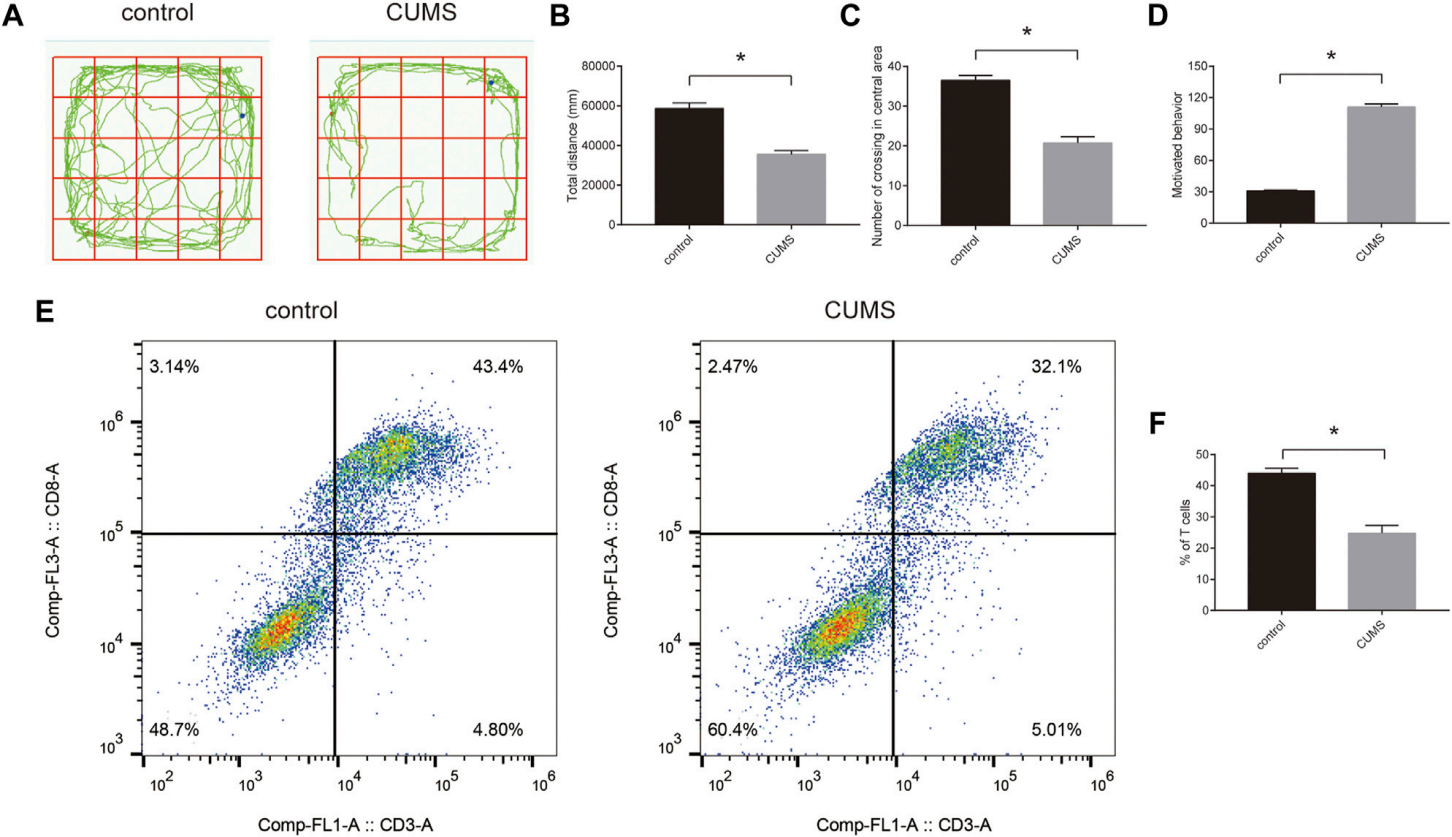
The proportion of CD8^+^ T cells in the peripheral blood of rats in the CUMS model. **(A–D)** The stress behavior was evaluated by open field experiment **(A–C)** and forced swimming test **(D)**. **(E,F)** The flow cytometric analysed the proportion of CD8^+^ T cells in the control groups and CUMS groups. Statistical analysis: unpaired t-tests. Data are presented as the mean ± SEM (*n* = 6 per group). *p < 0.05 compared to control. Control: stress-free group. CUMS, chronic mild unpredictable stress group.

To confirm the success of the chronic stress model, we first evaluated stress response by open field experiments and forced swimming tests. The results showed that rats exhibited stress response (Figures 7A–D). Furthermore, we detected the proportion of CD8^+^ T cells using flow cytometry. The results showed that the proportion of CD8^+^ T cells was reduced in the CUMS groups compared with the control groups (Figures 7E,F). In addition, we selected five key ARMGs reported in MDD for the first time from 10 key ARMGs for verification. qRT–PCR revealed significant decreases in the mRNA levels of Gnai3, Rab33b, Lamp2, and Kif5b in the CUMS groups compared to those in the related control groups (Supplementary Figure S7A–D).

## Discussion

In recent decades, evidence of immune imbalance in MDD has been increasing (Leonard, 2010). For example, compared with the healthy group, the immune cell counts in the whole blood of patients with MDD were increased, especially CD4^+^ T cells, neutrophils, and monocytes (Pfau et al., 2017). To further discuss the role of immune cell infiltration in MDD, we comprehensively evaluated immune infiltration by using CIBERSORT in MDD. In this study, we discovered different IIC proportions between peripheral blood from patients with MDD and that from the healthy group. Our results found that the proportions of resting NK cells, monocytes, and macrophages in patients with MDD were increased compared with those in the healthy group. The proportion of gamma delta T cells in patients with MDD was reduced compared with that in the healthy group. According to previous research reports, monocytes and macrophages are increased in MDD patients (Kronfol, 2002; Hughes et al., 2021), which is consistent with our results. Immune infiltration of resting gamma delta T cells and resting NK cells in MDD has not been reported.

Importantly, we found in GSE98793 dataset and GSE39653 dataset that the proportion of CD8^+^ T cells in peripheral blood of patients with MDD was lower than that of healthy people. We obtained the same results in the chronic stress rats model. CD8^+^ T cells play a major role in immune regulation. Recent data have demonstrated that reducing CD8 T lymphocyte apoptosis can regulate the immune microenvironment in depressed mice (Lu et al., 2017). Additionally, bipolar disorder is highly correlated with a reduction in circulating CD8^+^ T cell subpopulations (Magioncalda et al., 2018). Interestingly, although the proportion of CD8^+^ T cells was reduced in MDD patients, it was also different between patients with MDD. We found that MDD was divided into two subtypes—a high proportion of CD8^+^ T cells (T cells CD8H) and a low proportion of CD8^+^ T cells (T cells CD8L)—by further analysis, suggesting that the proportion of CD8^+^ T cells may be an essential factor affecting the development of depression. However, why some MDD samples had high CD8^+^ T cell infiltration while others did not is unclear. We further studied which biological function was related to the proportion of CD8 T cells in MDD patients.

Through functional enrichment analysis of the marker genes in each cluster, we found that the marker genes of the T cell CD8H cluster were enriched in terms related to autophagy. Autophagy can regulate immune system components, including natural killer (NK) cells, macrophages, dendritic cells (DCs), and T and B lymphocytes (Gerada and Ryan, 2020). In the process of macroautophagy, the autophagic vesicles that wrap abnormal proteins in the cytoplasm merge with lysosomes for degradation (Nishida et al., 2009). Interestingly, we found that the marker genes in the T cell CD8H cluster were significantly enriched in proteasome-mediated ubiquitin-dependent protein catabolic process, lysosomal membrane, ubiquitin-protein transferase activity, and ubiquitin-like protein transferase activity. Coincidentally, autopsies of MDD patients showed that synaptic protein synthesis in the prefrontal cortex was inhibited and mTOR phosphorylation levels were reduced, suggesting that autophagy was activated (Jernigan et al., 2011). Compelling evidence has revealed that autophagy is activated in MDD patients and that antidepressants exert antidepressant effects *via* autophagy regulation through different signalling pathways, suggesting that autophagy might be involved in the occurrence and development of depression (Shelton et al., 2011; Abelaira et al., 2014). According to some articles, markers for cellular autophagy are upregulated after antidepressant treatment (Zschocke and Rein, 2011). Additionally, the expression of markers for autophagy, such as beclin1, in the mouse brain increases after antidepressant treatment (Gassen et al., 2014). The above results indicate that autophagy pathways have vital effects on MDD. Interestingly, some articles suggest that mTOR (the major regulator of autophagy) is associated with memory CD8^+^ T cell differentiation. Based on these findings, rapamycin-mediated mTOR inhibition enhances memory CD8^+^ T cell magnitude and quality (Araki et al., 2009; Pearce et al., 2009). Therefore, autophagy plays an important role in the survival of effector CD8^+^ T cells (Xu et al., 2014). Our research confirmed that autophagy was highly correlated with the T cell CD8H cluster, suggesting that autophagy may be an important biological process that regulates the proportion of CD8^+^ T cells in MDD.

To date, although ARMGs have been widely reported as diagnostic and/or prognostic markers for many types of tumours, such as hepatocellular carcinoma (Huo et al., 2020), gastric cancer (Zhou et al., 2021), and ovarian cancer (Chen et al.), research on whether autophagy genes or proteins can serve as biomarkers in psychiatric disorders is limited. Recently, ARMGs have been reported to potentially be helpful for the diagnosis of schizophrenia (Li et al., 2021). However, the diagnostic performance of ARMGs for MDD has not yet been explored. We established a PPI network to investigate the correlations among ARMGs, which revealed 10 hub genes, namely, RAB1A, GNAI3, VAMP7, RAB33B, MYC, LAMP2, RAB11A, HIF1A, KIF5B, and PTEN. The subnetwork of VAMP7, MYC, RAB11A, HIF1A, and PTEN screened out from our study has been shown to play a role in MDD (Table 2). In particular, we discovered for the first time that RAB1A, GNAI3, RAB33B, LAMP2, and KIF5B might be potential markers for the diagnosis of depression. Rab1a controls the initiation of autophagy by regulating the trafficking of the ULK1 autophagy initiation complex to the phagophore (Webster et al., 2016). Its overexpression can increase the expression level of TLR4 (Song et al., 2020). GNAI3 has been recognized as a regulator of autophagy (Vural et al., 2013); it is abundantly expressed in immune cells and regulates cytokine responses to bacterial products (Li et al., 2019). Defective mitophagy is driven by dysregulation of KIF5B, which induces an NLRP3-dependent proinflammatory response (Yang et al., 2014). Notably, Kif5b deficiency within haematopoietic cells plays an important role in the anticancer response mediated by effective CD8^+^ T cells (Belabed et al., 2020). This evidence suggests that Rab1a, GNAI3, and KIF5B can regulate both autophagy and inflammation. At the same time, these autophagy-related genes may be markers explaining why some MDD patients have a high proportion of CD8 T cells. Whether these genes can change the proportion of CD8 T cells and exert antidepressant effects remains to be further experimentally verified.

## Limitation

This study had limitations. First, we identified several genes from microarray data analysis and verified their differential expression in the CUMS model. However, we did not further verify the functions of these selected genes. Therefore, we need a large number of clinical samples to verify our findings and clarify the potential mechanisms of how these genes affect the pathological stage.

## Conclusion

MDD patients may be clustered according to the proportion of CD8^+^ T cells, which corresponds to the diagnosis for a certain type of patient. After clustering, the marker genes of the T cell CD8H cluster were found to be significantly related to autophagy, suggesting that autophagy may affect the immune microenvironment of MDD patients. We also screened five hub autophagy-related genes, which may offer new hope for the treatment and diagnosis of MDD patients in the future, although further study is required.

## Data Availability

The datasets presented in this study can be found in online repositories. The names of the repository/repositories and accession number(s) can be found in the article/Supplementary Material.
