# Supplementary material for: Identification of Potential Diagnoses Based on Immune Infiltration and Autophagy Characteristics in Major Depressive Disorder
Source: Front Genet. 2022 Apr 26;13:702366. doi: 10.3389/fgene.2022.702366 (PMC9087348; doi:10.3389/fgene.2022.702366)
Supplement: Supplementary file 1 [file DataSheet1.docx]

**Supplementary Material**


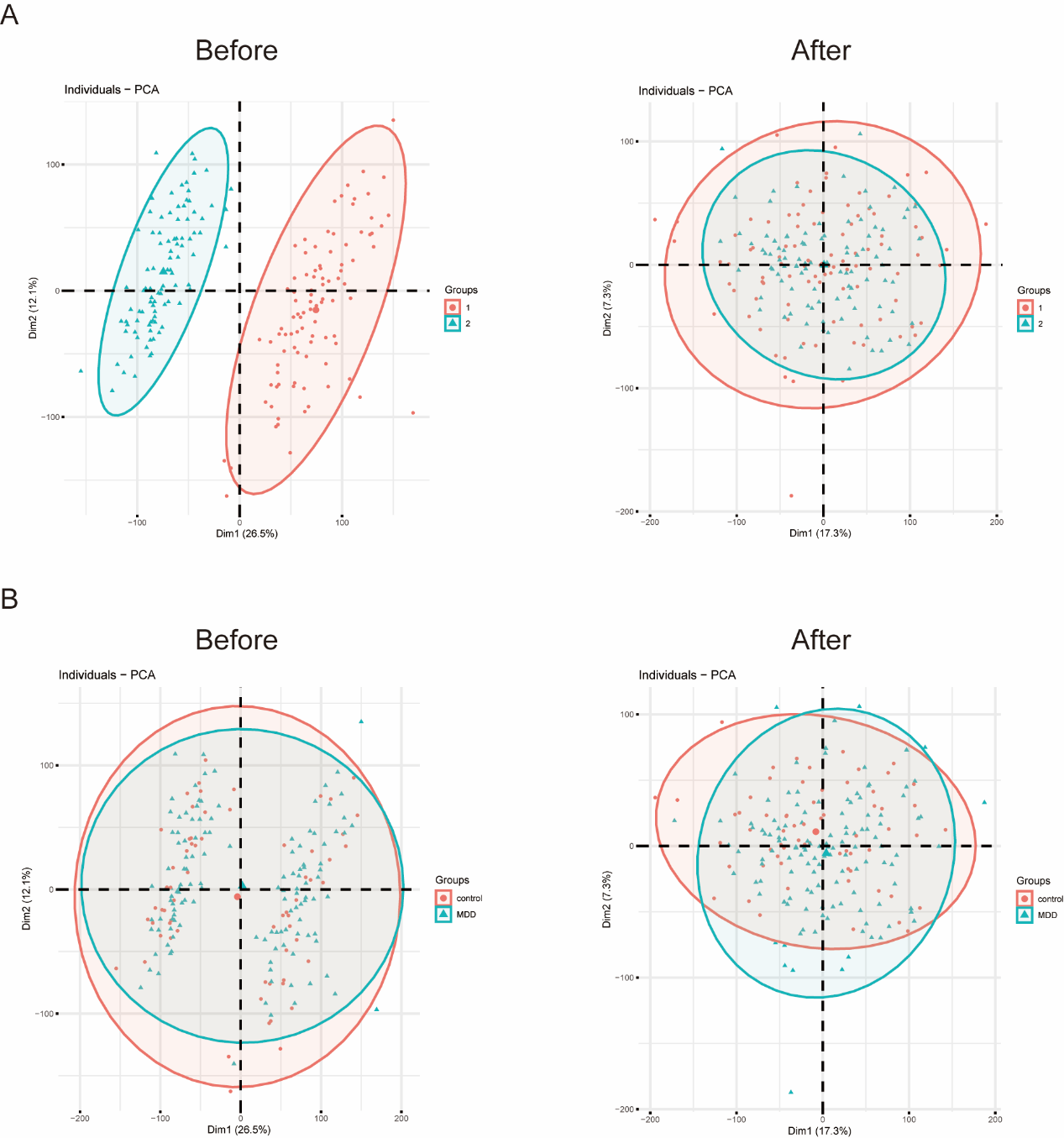


**SUPPLEMENTARY FIGURE 1.** Data processing in the GSE98793 dataset. (A, B) The batch effects between different batches of sequencing data (A), between MDD samples and control samples (B), were analyzed by PCA.


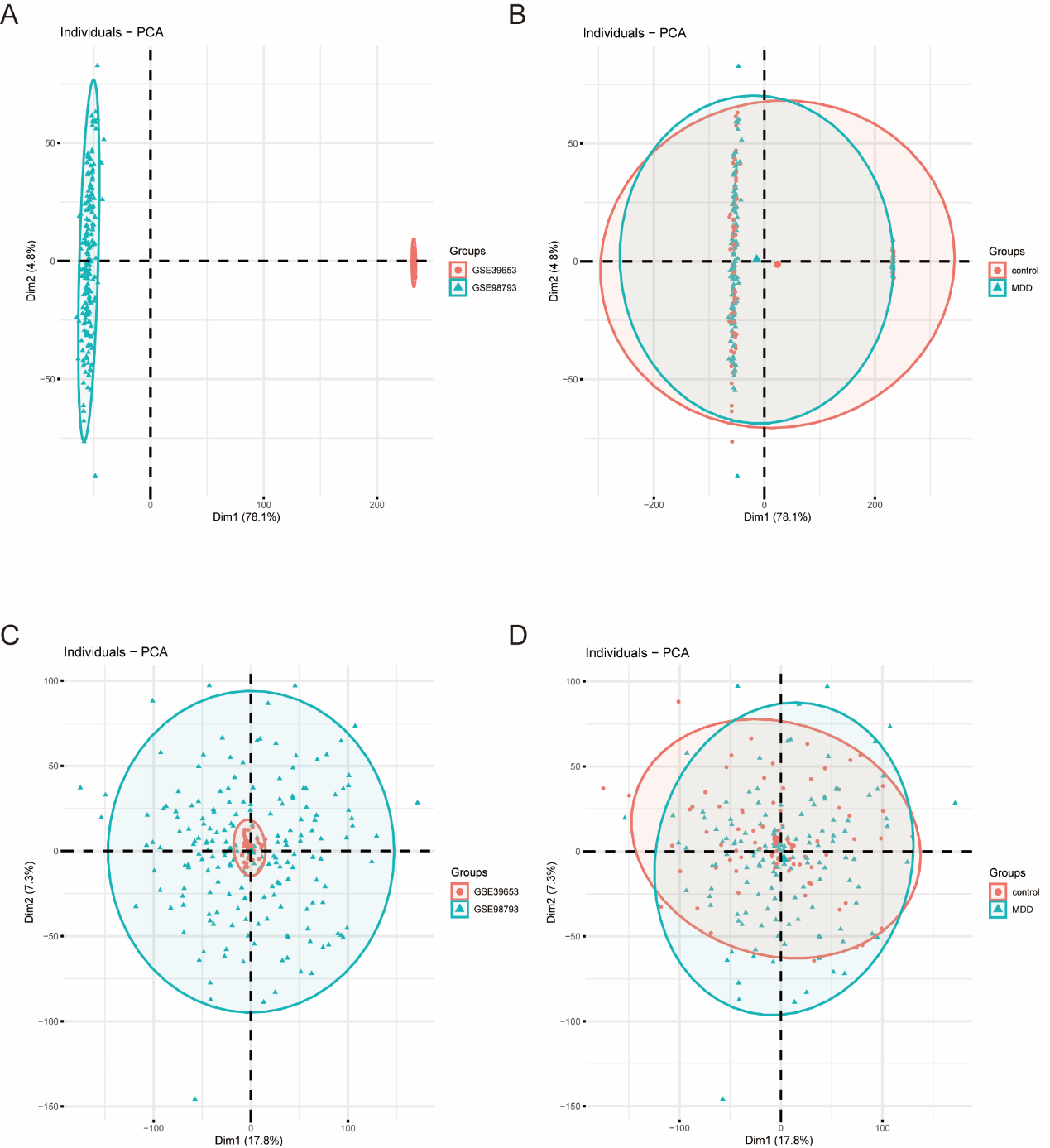


**SUPPLEMENTARY FIGURE 2.** Data processing in the GSE98793 dataset and GSE39653 dataset. (A-D) The batch effects between GSE39653 and GSE98793 datasets, and between MDD samples and control samples, were analyzed by PCA.


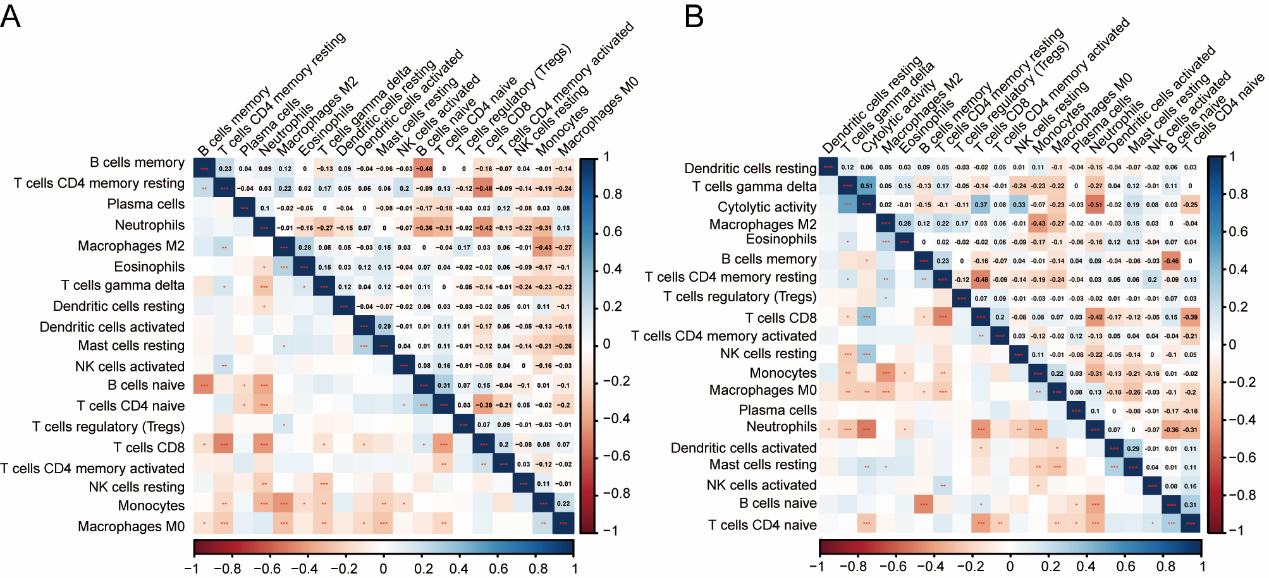


**SUPPLEMENTARY FIGURE 3.** Analysis of immune landscape in MDD. (A) Correlation matrix of immune cell proportions. (B) Immune cytolytic activity of immune cell proportions.


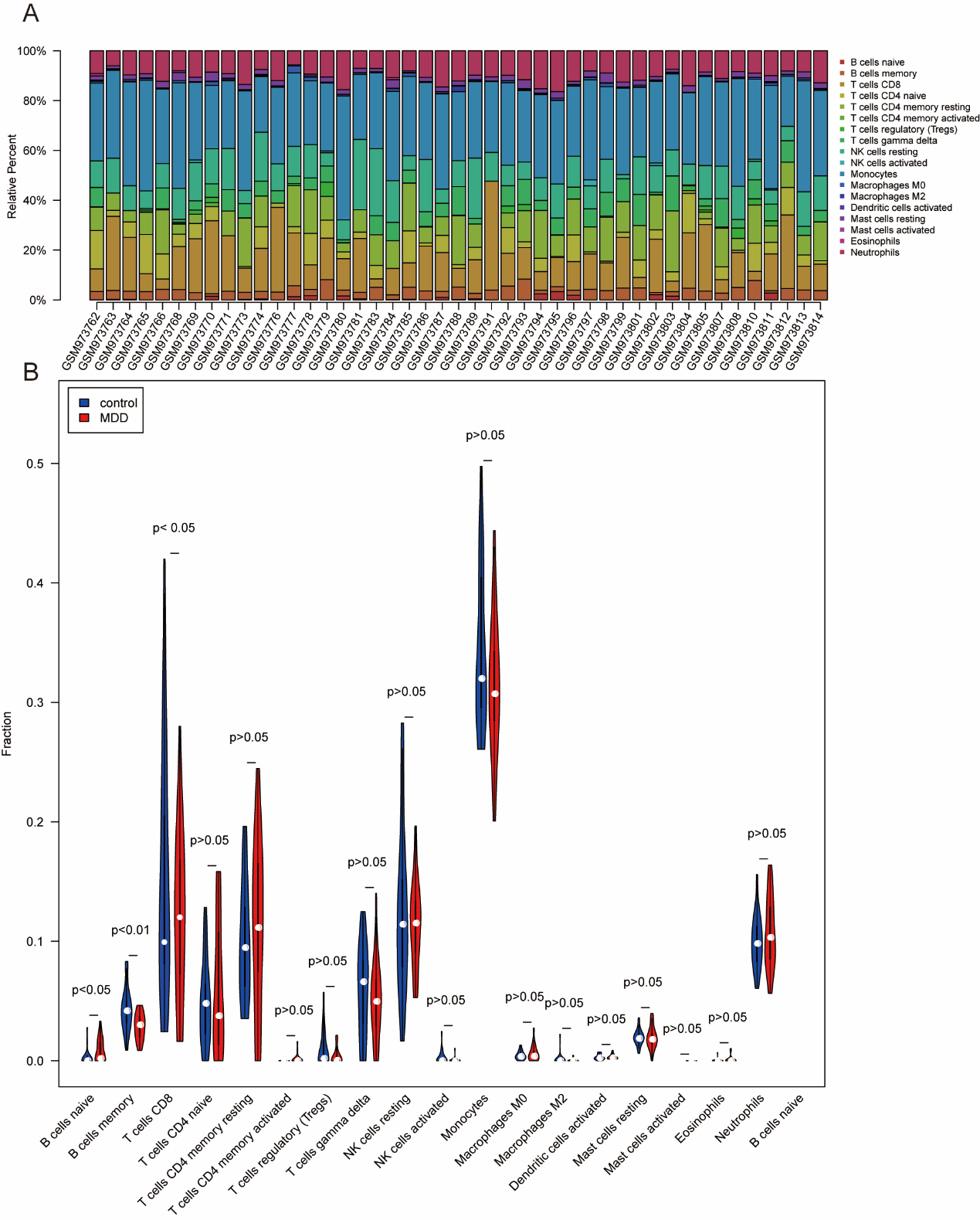


**SUPPLEMENTARY FIGURE 4.** The landscape of immune infiltration between control and MDD samples in the GSE39653 dataset. (A) The relative percentage of 22 subpopulations of immune cells in 53 samples from GSE39653 dataset. (B) The difference of immune infiltration between MDD and control (The control group was marked as blue color and MDD group was marked as red color. *P* values < 0.05 were considered as statistical significance).

**
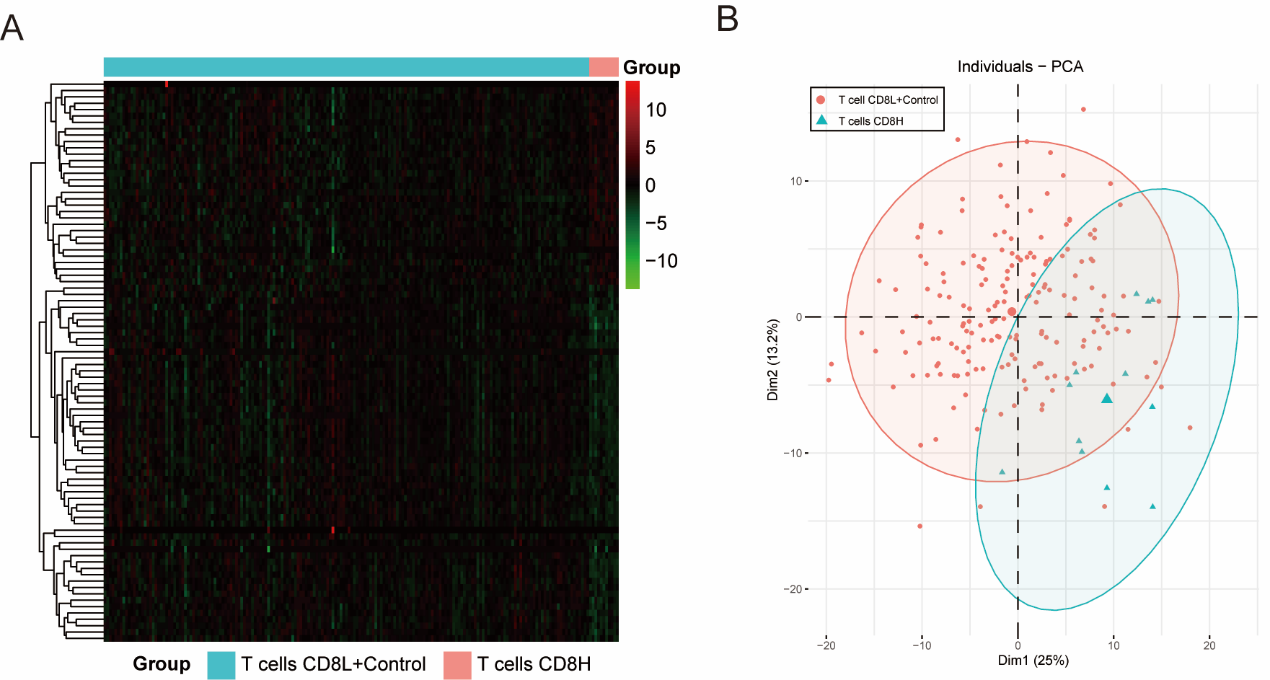
**

**SUPPLEMENTARY FIGURE 5.** Identification of ARMGs in the T cell CD8H cluster. **(A)** The heatmap of 220 ARMGs in T cell CD8H cluster (presented in pink color) and other (normal+T cell CD8L; presented in blue-green color) cluster. Red: higher expression; green: lower expression. **(B)** PCA analysis of the subgroup of T cell CD8H and other (control+T cell CD8L). Red dots indicate the other samples and blue-green triangles indicate T cell CD8H samples. ARMGs, autophagy-related marker genes; DEG, differentially expressed gene; ARGs, autophagy-related genes; HADb, Human Autophagy Database. Slightly larger circles/triangles represent the center of sample distribution in the corresponding group, e.g., a slightly larger red circle represents the center of distribution of all T cell CD8L+control group samples.


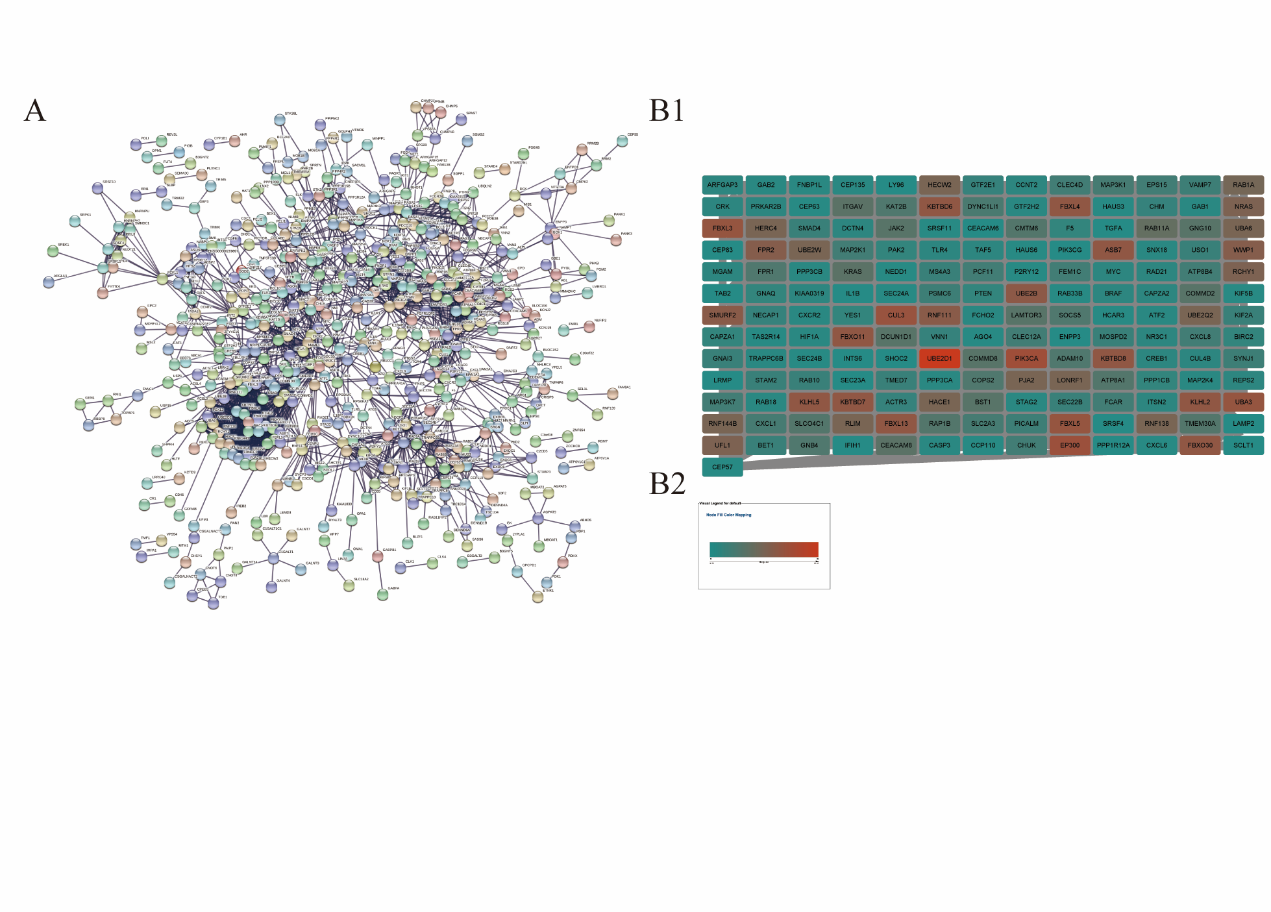


**SUPPLEMENTARY FIGURE 6.** PPI network analysis. (A) The PPI network was analyzed by STRING software (interaction score < 0.09). Here were 566 nodes and 2299 edged in the PPI network. (B) PPI network with 170 nodes and 1363 edges was constructed using the degree value ≥ 10 by STRING. PPI, protein-protein interaction.


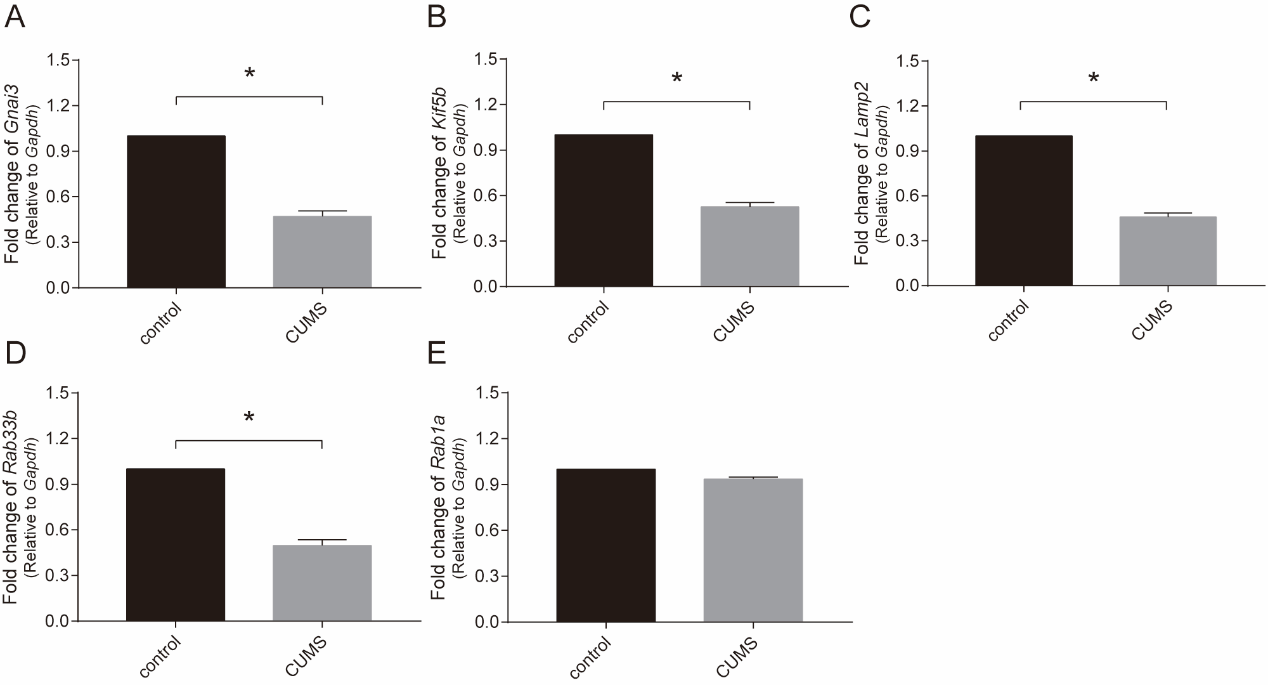


**SUPPLEMENTARY FIGURE 7.** The level of corresponding key genes in the peripheral blood of rats in the CUMS model**.** (A-E) qRT-PCR results showing the mRNA levels of *Rab1a*, *Gnai3*, *Rab33b*, *Lamp2*, and *Kif5b* among the control and CUMS groups. The mean values in control samples were set to 1.0. Data are presented as mean ± SEM (n = 6/group). *p < 0.05 compared to control.


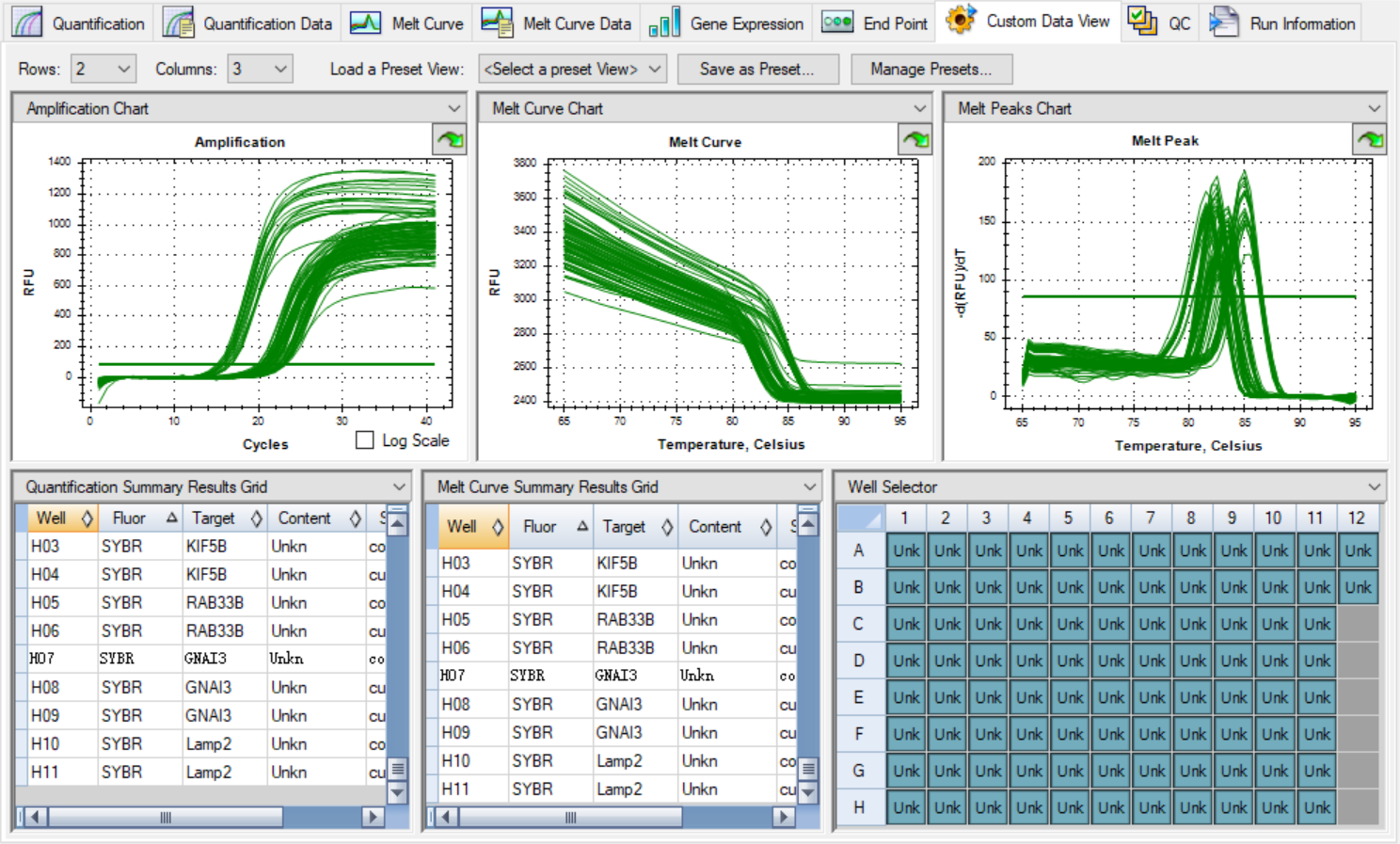


**SUPPLEMENTARY FIGURE 8.** Partial melt curve and amplification curve in PCR experiment

**SUPPLEMENTARY TABLE S1.** Comparison of CIBERSORT immune fractions between MDD samples and controls in the GSE98793 dataset.

|  |  | controlmean | controsd | MDDmean | MDDsd | pvalue | labels |
| --- | --- | --- | --- | --- | --- | --- | --- |
| 1 | B cells naive | 0.0094 | 0.0105 | 0.0093 | 0.0089 | *p* > 0.05 | *p* > 0.05 |
| 2 | B cells memory | 0.0088 | 0.0093 | 0.0081 | 0.0087 | *p* > 0.05 | *p* > 0.05 |
| 3 | Plasma cells | 0.0002 | 0.0005 | 0.0004 | 0.0019 | *p* > 0.05 | *p* > 0.05 |
| 4 | **T cells CD8** | 0.0925 | 0.0733 | 0.0745 | 0.0721 | *p* < 0.05 | *p* < 0.05 |
| 5 | T cells CD4 naive | 0.1196 | 0.0548 | 0.1177 | 0.0474 | *p* > 0.05 | *p* > 0.05 |
| 6 | T cells CD4 memory resting | 0.0282 | 0.0326 | 0.0241 | 0.0306 | *p* > 0.05 | *p* > 0.05 |
| 7 | T cells CD4 memory activated | 0.0195 | 0.0140 | 0.0199 | 0.0119 | *p* > 0.05 | *p* > 0.05 |
| 8 | T cells regulatory (Tregs) | 0.0001 | 0.0005 | 0.0000 | 0.0004 | *p* > 0.05 | *p* > 0.05 |
| 9 | **T cells gamma delta** | 0.0845 | 0.0485 | 0.0590 | 0.0375 | *p* < 0.001 | p<0.001 |
| 10 | **NK cells resting** | 0.0909 | 0.0361 | 0.1050 | 0.0328 | *p* < 0.01 | *p* < 0.01 |
| 11 | NK cells activated | 0.0000 | 0.0000 | 0.0001 | 0.0007 | *p* > 0.05 | *p* > 0.05 |
| 12 | **Monocytes** | 0.0719 | 0.0362 | 0.0877 | 0.0366 | *p* < 0.01 | *p* < 0.01 |
| 13 | **Macrophages M0** | 0.0025 | 0.0085 | 0.0060 | 0.0118 | *p* < 0.01 | *p* < 0.01 |
| 14 | Macrophages M2 | 0.0035 | 0.0036 | 0.0031 | 0.0039 | *p* > 0.05 | *p* > 0.05 |
| 15 | Dendritic cells resting | 0.0002 | 0.0006 | 0.0001 | 0.0004 | *p* > 0.05 | *p* > 0.05 |
| 16 | Dendritic cells activated | 0.0008 | 0.0015 | 0.0008 | 0.0015 | *p* > 0.05 | *p* > 0.05 |
| 17 | Mast cells resting | 0.0160 | 0.0093 | 0.0154 | 0.0099 | *p* > 0.05 | *p* > 0.05 |
| 18 | Eosinophils | 0.0077 | 0.0115 | 0.0060 | 0.0111 | *p* > 0.05 | *p* > 0.05 |
| 19 | Neutrophils | 0.4438 | 0.0765 | 0.4628 | 0.0826 | *p* > 0.05 | *p* > 0.05 |

**SUPPLEMENTARY TABLE S2.** Comparison of CIBERSORT immune fractions between MDD samples and controls in the GSE39653 dataset.

|  |  | **controlmean** | **controsd** | **MDDmean** | **MDDsd** | **pvalue** |
| --- | --- | --- | --- | --- | --- | --- |
| 1 | **B cells naive** | 0.0025 | 0.0064 | 0.0083 | 0.0101 | *p* < 0.05 |
| 2 | **B cells memory** | 0.0431 | 0.0183 | 0.0286 | 0.0113 | *p* < 0.01 |
| 3 | **T cells CD8** | 0.1783 | 0.0702 | 0.1134 | 0.0340 | *p* < 0.05 |
| 4 | T cells CD4 naive | 0.0489 | 0.0353 | 0.0597 | 0.0554 | *p* > 0.05 |
| 5 | T cells CD4 memory resting | 0.1001 | 0.0492 | 0.1097 | 0.0719 | *p* > 0.05 |
| 6 | T cells CD4 memory activated | 0.0001 | 0.0004 | 0.0012 | 0.0039 | *p* > 0.05 |
| 7 | T cells regulatory (Tregs) | 0.0097 | 0.0154 | 0.0039 | 0.0068 | *p* > 0.05 |
| 8 | T cells gamma delta | 0.0592 | 0.0384 | 0.0511 | 0.0335 | *p* > 0.05 |
| 9 | NK cells resting | 0.1259 | 0.0687 | 0.1133 | 0.0364 | *p* > 0.05 |
| 10 | NK cells activated | 0.0023 | 0.0056 | 0.0005 | 0.0023 | *p* > 0.05 |
| 11 | Monocytes | 0.3426 | 0.0657 | 0.3133 | 0.0575 | *p* > 0.05 |
| 12 | Macrophages M0 | 0.0044 | 0.0035 | 0.0061 | 0.0067 | *p* > 0.05 |
| 13 | Macrophages M2 | 0.0010 | 0.0045 | 0.0004 | 0.0012 | *p* > 0.05 |
| 14 | Dendritic cells activated | 0.0030 | 0.0021 | 0.0032 | 0.0020 | *p* > 0.05 |
| 15 | Mast cells resting | 0.0197 | 0.0064 | 0.0194 | 0.0093 | *p* > 0.05 |
| 16 | Mast cells activated | 0.0000 | 0.0000 | 0.0000 | 0.0001 | *p* > 0.05 |
| 17 | Eosinophils | 0.0003 | 0.0014 | 0.0009 | 0.0028 | *p* > 0.05 |
| 18 | Neutrophils | 0.0990 | 0.0215 | 0.1069 | 0.0290 | *p* > 0.05 |

**SUPPLEMENTARY TABLE S3.** GO enrichment analyses of ARMGs in T cells CD8H cluster.

| **ID** | **Description** | **Counts** | **pValue** | **FDR** | **Category** |
| --- | --- | --- | --- | --- | --- |
| GO:0016236 | macroautophagy | 36 | *p* < 0.0001 | *p* < 0.0001 | GO_BP |
| GO:0061919 | process utilizing autophagic mechanism | 40 | *p* < 0.0001 | *p* < 0.0001 | GO_BP |
| GO:0000045 | autophagosome assembly | 16 | *p* < 0.0001 | *p* < 0.0001 | GO_BP |
| GO:1905037 | autophagosome organization | 16 | *p* < 0.0001 | *p* < 0.0001 | GO_BP |
| GO:0007033 | vacuole organization | 18 | *p* < 0.0001 | *p* < 0.0001 | GO_BP |
| GO:0016241 | regulation of macroautophagy | 17 | *p* < 0.0001 | *p* < 0.0001 | GO_BP |
| GO:0031668 | cellular response to extracellular stimulus | 19 | *p* < 0.0001 | *p* < 0.0001 | GO_BP |
| GO:0010506 | regulation of autophagy | 20 | *p* < 0.0001 | *p* < 0.0001 | GO_BP |
| GO:0071496 | cellular response to external stimulus | 20 | *p* < 0.0001 | *p* < 0.0001 | GO_BP |
| GO:0031667 | response to nutrient levels | 23 | *p* < 0.0001 | *p* < 0.0001 | GO_BP |
| GO:0005776 | autophagosome | 12 | *p* < 0.0001 | *p* < 0.0001 | GO_CC |
| GO:0000407 | phagophore assembly site | 8 | *p* < 0.0001 | *p* < 0.0001 | GO_CC |
| GO:0098589 | membrane region | 14 | *p* < 0.0001 | *p* < 0.0001 | GO_CC |
| GO:0045335 | phagocytic vesicle | 9 | *p* < 0.0001 | *p* < 0.0001 | GO_CC |
| GO:0000421 | autophagosome membrane | 6 | *p* < 0.0001 | *p* < 0.0001 | GO_CC |
| GO:0030670 | phagocytic vesicle membrane | 7 | *p* < 0.0001 | *p* < 0.0001 | GO_CC |
| GO:0016234 | inclusion body | 6 | *p* < 0.0001 | *p* < 0.0001 | GO_CC |
| GO:0030176 | integral component of endoplasmic reticulum membrane | 7 | *p* < 0.0001 | *p* < 0.001 | GO_CC |
| GO:0032838 | plasma membrane bounded cell projection cytoplasm | 8 | *p* < 0.0001 | *p* < 0.001 | GO_CC |
| GO:0031143 | pseudopodium | 3 | *p* < 0.0001 | *p* < 0.001 | GO_CC |
| GO:0031072 | heat shock protein binding | 11 | *p* < 0.0001 | *p* < 0.0001 | GO_MF |
| GO:0004197 | cysteine-type endopeptidase activity | 9 | *p* < 0.0001 | *p* < 0.0001 | GO_MF |
| GO:0044389 | ubiquitin-like protein ligase binding | 11 | *p* < 0.0001 | *p* < 0.0001 | GO_MF |
| GO:0051087 | chaperone binding | 6 | *p* < 0.0001 | *p* < 0.001 | GO_MF |
| GO:0030291 | protein serine/threonine kinase inhibitor activity | 4 | *p* < 0.0001 | *p* < 0.001 | GO_MF |
| GO:0051082 | unfolded protein binding | 6 | *p* < 0.0001 | *p* < 0.01 | GO_MF |
| GO:0045296 | cadherin binding | 9 | *p* < 0.0001 | *p* < 0.01 | GO_MF |
| GO:0004674 | protein serine/threonine kinase activity | 10 | *p* < 0.0001 | *p* < 0.01 | GO_MF |
| GO:0000149 | SNARE binding | 5 | *p* < 0.001 | *p* < 0.01 | GO_MF |
| GO:0051787 | misfolded protein binding | 3 | *p* < 0.001 | *p* < 0.01 | GO_MF |

**SUPPLEMENTARY TABLE S4.** KEGG pathway enrichment analysis of ARMGs in T cells CD8H cluster.

| **ID** | **Description** | **Counts** | **pValue** | **FDR** |
| --- | --- | --- | --- | --- |
| hsa04140 | Autophagy - animal | 29 | *p* < 0.0001 | *p* < 0.0001 |
| hsa04136 | Autophagy - other | 13 | *p* < 0.0001 | *p* < 0.0001 |
| hsa05167 | Kaposi sarcoma-associated herpesvirus infection | 16 | *p* < 0.0001 | *p* < 0.0001 |
| hsa05163 | Human cytomegalovirus infection | 16 | *p* < 0.0001 | *p* < 0.0001 |
| hsa04211 | Longevity regulating pathway | 11 | *p* < 0.0001 | *p* < 0.0001 |
| hsa05131 | Shigellosis | 16 | *p* < 0.0001 | *p* < 0.0001 |
| hsa04141 | Protein processing in endoplasmic reticulum | 13 | *p* < 0.0001 | *p* < 0.0001 |
| hsa04218 | Cellular senescence | 12 | *p* < 0.0001 | *p* < 0.0001 |
| hsa05212 | Pancreatic cancer | 9 | *p* < 0.0001 | *p* < 0.0001 |
| hsa04210 | Apoptosis | 11 | *p* < 0.0001 | *p* < 0.0001 |
